# Nerve block or wound infiltration with liposomal bupivacaine for postoperative pain management in open orthopedic surgery: a meta-analysis of 38 randomized controlled trials involving 3,389 patients

**DOI:** 10.3389/fmed.2026.1820439

**Published:** 2026-09-18

**Authors:** Jinsheng Yu, Zixing Zhao, Yan Lu, Jinda Dong, Wenjun Chen, Xiaodong Wei, Jiahong Nong, Chenglong Wang, Jianxun Wei, Zhuhai Li, Xiaoping Mu

**Affiliations:** 1 Department of Joint Surgery and Sports Medicine, The Sixth People's Hospital of Huizhou, Affiliated Huiyang Hospital of Southern Medical University, Huizhou, China; 2 Department of Spine Surgery, The People's Hospital of Guangxi Zhuang Autonomous Region, Nanning, China; 3 Guangxi Academy of Medical Sciences, Nanning, China; 4 Department of Blood Transfusion, The People's Hospital of Guangxi Zhuang Autonomous Region, Nanning, China; 5 Department of Spine Surgery, The Guangxi Corps Hospital of the Chinese People's Armed Police Force, Nanning, China; 6 Department of Orthopedics, Taizhou Hospital of Zhejiang Province Affiliated to Wenzhou Medical University, Taizhou, China

**Keywords:** liposomal bupivacaine, local anesthesia, opioid, pain management, shoulder replacement, spinal fusion, total hip arthroplasty, total knee arthroplasty

## Abstract

**Background:**

Patients undergoing open orthopedic surgeries often report substantial postoperative pain. The present work aimed to provide effective evidence for perioperative pain management in open orthopedic surgeries through comprehensive electronic database searches and the inclusion of more high-quality randomized controlled trials (RCTs) that report on the safety and effectiveness of liposomal bupivacaine (LB) in these surgeries.

**Methods:**

We performed the present work strictly under the guidelines of the Cochrane handbook and PRISMA recommendations. Under the guidance of the established retrieval strategy, two researchers independently performed the literature retrieval. Each included study was assessed in according with Cochrane risk of bias tool. We utilized STATA software to perform all meta-analyses in the current study.

**Results:**

38 RCTs encompassing 3,389 patients were included in this study. The results demonstrated that there was statistical significance in pain intensity scores on POD2 [SMD = −0.25, 95% CI (−0.49 to −0.02)] and a trend toward statistical significance on postoperative day 1 (POD1) [SMD = −0.17, 95% CI (−0.35 to 0.00)] between the two groups. However, no statistical differences in pain scores on POD0, POD3, opioid consumption (total, POD1, POD2, POD3), adverse events, patient satisfaction, and the length of hospital stay.

**Conclusion:**

Compared to conventional medications, LB does not demonstrate a significant advantage in perioperative pain management, nor does it significantly reduce opioid consumption. Further high-quality RCTs are required to confirm these findings and greater attention should be given to the long-term safety of LB to ensure its reliability for practical use.

## 1 Introduction

Surgical procedures involving the musculoskeletal system emerge as one of the most prevalent and painful procedures in clinical practice (1). A survey released in 2013 (2) revealed that 22 of the 40 surgical procedures with the highest-rated pain were related to orthopedic or trauma surgeries of the extremities. Despite the advancements in perioperative pain control, patients undergoing open orthopedic surgeries, including but not limited to arthroplasty, lumbar fusion, and fracture surgery, still report substantial postoperative pain (3). A cross-sectional observational study (4) involving over 15,000 surgical patients in the UK reported that 11% of patients experienced severe pain, and 37% reported moderate pain within the first 24 h after surgery. Moreover, approximately 47% of patients undergoing total hip arthroplasty (THA) and 58% undergoing total knee arthroplasty (TKA) experienced moderate to severe pain on the first postoperative day (5).

Acute pain following orthopedic surgery can result in a variety of detrimental effects, such as delayed postoperative recovery, prolonged hospital stays, increased postoperative complications, limited rehabilitation participation, prolonged reliance on opioids, and even the development of chronic pain (6). Postoperative pain after open orthopedic surgery is an unfavorable prognostic factor (7). Consequently, postoperative pain management has emerged as a critical component of the healthcare team's responsibilities. Attention and proactive efforts in perioperative pain prevention and management strategies are imperative (8), allowing patients to traverse the postoperative recovery phase smoothly.

Opioids have traditionally been the most frequently prescribed category of pain-killer drugs during the perioperative period in orthopedic surgery (9). Over the past decade, opioid-related costs in the United States have surged dramatically, with an increase of up to an astonishing 660% (10). Although opioids are effective in pain control, their well-known issues with abuse, addiction, and even overdose deaths are significant concerns (11–13). To reduce unnecessary opioid use, multimodal analgesia approaches have gradually gained acceptance and popularity in clinical practice. Specifically, orthopedic surgeons increasingly emphasize the utilization of local anesthetics for regional blocks or wound infiltration as part of a multimodal analgesia strategy.

Liposome bupivacaine (LB) is a multi-vesicular liposome formulation containing bupivacaine that is approved for wound infiltration and regional nerve block. This formulation achieves analgesia for over 72 h through a sustained and slow release of bupivacaine (14). Currently, research on the use of LB in perioperative pain control for orthopedic surgery has yielded inconsistent results. Previous studies (15, 16) have reported that LB effectively reduces postoperative pain, minimizing the need for rescue analgesics, and mitigates opioid-related adverse effects after orthopedic surgeries. However, several studies (17, 18) have failed to demonstrate any significant advantage of LB compared to traditional analgesic drugs or non-opioid alternatives.

Previous meta-analyses (19, 20) have reported on the use of LB during the perioperative period for certain orthopedic procedures. However, a consistent pattern among the conclusions drawn by various studies has been elusive, with evidence strength varying accordingly. Nowadays, more studies on the local administration of LB in open orthopedic surgeries have been published. These fresh insights may bridge the previously identified discrepancies in research outcomes. Furthermore, given that using higher-cost LB in clinical practice is inappropriate without confirmed clinical benefits (21), its application in total joint arthroplasty has also shown a downward trend (22). The present work aims to analyze more published studies on the local application of LB during the perioperative period following open orthopedic surgeries, addressing previous gaps and producing findings that are backed by a higher level of evidence.

## 2 Materials and methods

The present work was a comprehensive secondary literature review focusing on the published randomized controlled trials (RCTs) exploring the local administration of LB in open orthopedic surgeries. It did not involve any ethical issues related to human or animal subjects. Therefore, the research protocol has been exempted from approval by our institution's ethics committee. We performed the present work strictly under the execution standards of systematic review as outlined in the Cochrane manual (23). The reporting of our findings conformed to the guidelines of the Preferred Reporting Items for Systematic Reviews and Meta-Analyses (PRISMA) recommended by the PRISMA work group (24). The protocol of the present work was prospectively registered with the International Prospective Register of Systematic Reviews (registration number CRD42024618098).

### 2.1 Literature search strategy and data sources

The literature retrieval strategy for this meta-analysis was developed after multiple trial electronic searches conducted by two researchers with over 3 years of experience in literature retrieval. Under the guidance of the established retrieval strategy, we employed Boolean operators “AND,” “OR,” and “NOT” to effectively combine the keywords “liposomal bupivacaine”, “liposome”, “exparel”, with other keywords “orthopedic surgery”, “spine”, “arthroplasty”, “joint replacement”, “shoulder surgery”, “fracture”. The following electronic databases were searched: Medline via PubMed, Web of Science, Embase, Cochrane Central Register of Controlled Clinical Trials, and Google Scholar. The search strategy for each database is detailed in Supplementary File 1. To ensure a comprehensive search, the present work imposed no language restrictions on the published studies, and the timeframe for literature retrieval was set from January 2010 to September 2025. Furthermore, we manually searched for the studies included in the present work and references from reviews or meta-analyses under the same or similar topic to ensure comprehensive literature retrieval.

### 2.2 Eligibility criteria

To directly address the clinical questions this study aims to clarify, we used the Population, Intervention, Comparison, Outcomes, and Study (PICOS) framework to establish the inclusion criteria for the present work. (1) Population: adult patients who received any open orthopedic surgeries (including but not restricted to hip/knee replacement, spinal surgery, extremity fracture surgery, etc.) with a peripheral field or nerve block. (2) Intervention: local administration of LB. (3) Comparison: placebo (saline or nothing) or any local analgesia (bupivacaine, ropivacaine, etc.), combined or not with perineural or subarachnoid adjuncts. (4) Outcomes: patient-reported pain scores (including all types of pain scales with standardization of pain intensity), opioid consumption, and adverse events (including the incidence of nausea or vomiting, dizziness, constipation, pruritus). (5) Study design: RCT, combined or not with the blind.

### 2.3 Study selection

Two researchers independently screened the literature and checked with each other to guarantee the accuracy of the screening process. For any disputed records, a consensus was reached through internal discussion, with the introduction of a third senior researcher if necessary to resolve the disagreements. All records from electronic databases were first imported into literature management tools to remove duplicate literature. During the initial screening, two researchers assessed the relevance of the studies by reading the titles and abstracts, then labeled each record as “include,” “exclude,” or “uncertain”. For records marked as “include” or “uncertain,” the full texts were downloaded and reviewed to determine whether they met the inclusion criteria for the current work.

### 2.4 Data extraction

Two researchers independently extracted the data and entered it into the corresponding fields of a pre-designed standardized electronic data collection form. For studies with missing, incomplete, or inadequately formatted data unsuitable for statistical analysis, we would email the authors or study sponsors. Given the controversy over estimating data from multiple scoring standards, especially those with small sample sizes (25), we did not perform data estimation in this meta-analysis. In this meta-analysis, we extracted the following data from each included study: (1) Study characteristics: first author name, study location, publication year, study design, surgical approach, and patient baseline characteristics. (2) Intervention: drug, dose, mode of administration, duration. (3) Outcome indicators: postoperative pain intensity scores, opioid consumption, patient satisfaction, length of hospital stay, and treatment-emergent adverse events.

### 2.5 Risk of bias assessment

The present study only included published RCTs that investigated the local application of LB in open orthopedic surgeries. Two researchers who have received training in literature quality evaluation independently evaluated the included studies using the Cochrane risk of bias tool. Each included RCT was required to respond to “high risk,” “low risk,” or “unclear” for the seven domains of random sequence generation, allocation concealment, blinding of study participants, blinded outcomes assessment, incomplete outcome data, selective reporting, and other biases (23). If more than two domains in an RCT were rated as “high risk,” the risk of bias for that study was defined as high (26). Once the evaluations are completed, the results obtained by both researchers are cross-checked and compared. Disagreements between the researchers were first resolved through internal negotiation. A third senior researcher was consulted to resolve the discrepancies if no consensus could be reached.

### 2.6 Statistical analysis

We utilized STATA software (Version 17, StataCorp LP, College Station, TX, USA) to perform all meta-analyses in the current study. The software estimates the weighted/standardized mean differences (W/SMD) for continuous data, and the risk ratio (RR) for binary data, with an overall estimate of the pooled effect. The *I*^2^ coefficient was employed to detect heterogeneity among studies, with pre-defined ranges to classify them as low (25%−49%), moderate (50%−74%), or high (≥75%) (27). We applied a random-effects model for results with moderate or high heterogeneity, whereas a fixed-effects model was used for results with low heterogeneity (28).

Taking into account the distinct features of the studies included in this meta-analysis, we surmised that the following factors might significantly impact heterogeneity: type of surgery (joint replacement, spinal surgery, fracture surgery), the dosage of LB (133 mg or 266 mg), interventions in the control group (plain bupivacaine, ropivacaine, or saline), administration technique (nerve block, local infiltration). We will conduct the subgroup analysis and meta-regression on the primary outcomes with high heterogeneity based on these factors to explore the drivers of heterogeneity.

We performed the sensitivity analysis using a stepwise exclusion method to verify the robustness of the pooled results for outcome measures. We have employed the Egger's test to detect potential publication bias. The results of this meta-analysis would be presented as W/SMD or RR, with 95% confidence intervals (CIs). A two-tailed *P*-value of < 0.05 was considered statistically significant.

## 3 Results

Among the initially retrieved 2,827 articles, we excluded 1,225 duplicate studies. Subsequently, 1,471 articles were further excluded by reviewing the titles and abstracts. Among the remaining 131 articles, we carefully selected 38 studies (16, 21, 29–64) for inclusion in our current work after thoroughly reading their full texts. The process of literature retrieval is depicted in Figure 1.

**Figure 1:**
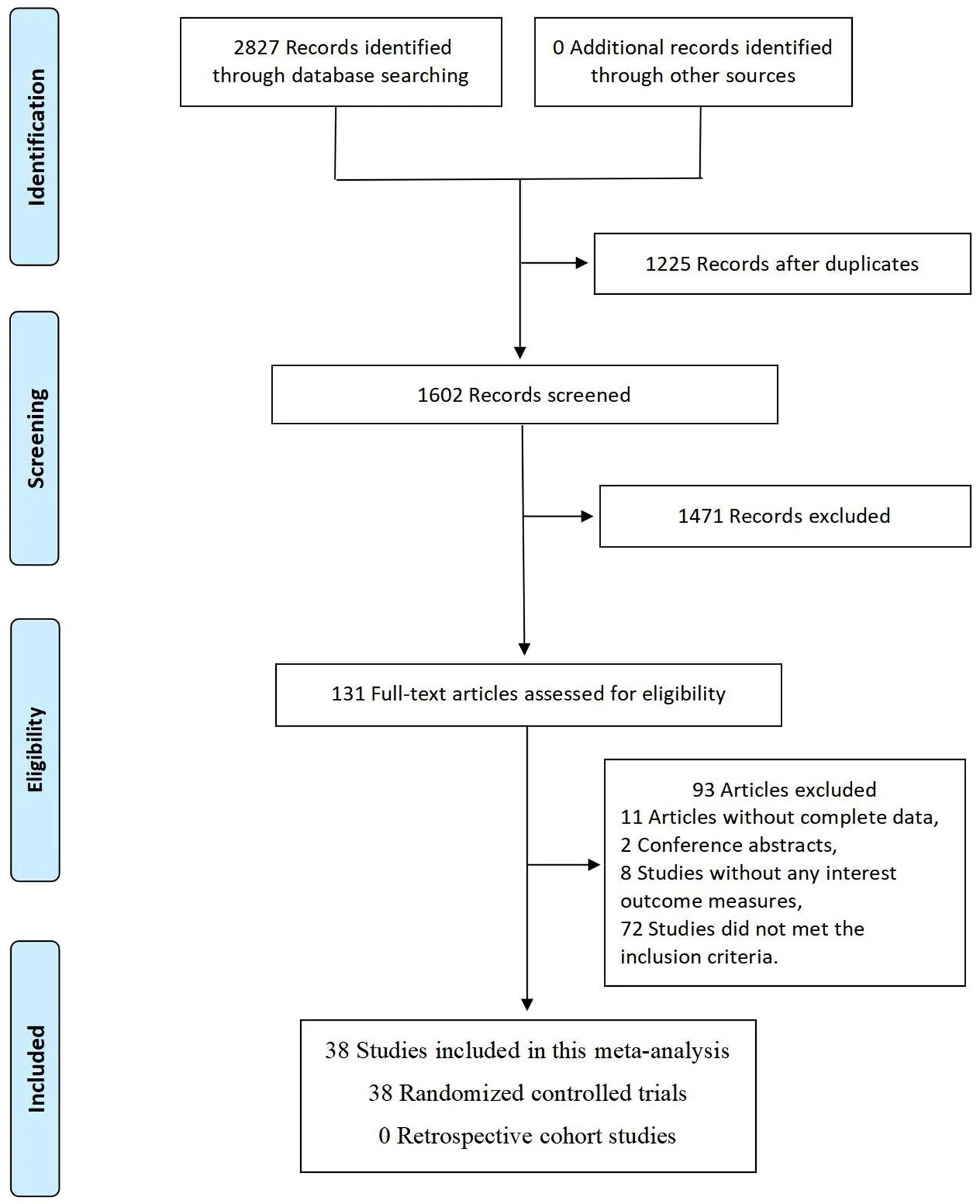
Flow diagram of study selection.

The overall risk of bias was assessed as low in 8 trials, unclear in 25 trials, and high in 5 trials. In the five studies rated as high risk, blinding of study participants and blinded outcome assessment emerged as the primary risk domains according to the seven-domain framework of the Cochrane risk of bias tool. In contrast, within the 25 studies rated as unclear risk, selective reporting (15 studies) and other biases (19 studies) emerged as the predominant risk domains. Figure 2 summarizes a comprehensive overview of the bias risk for each included study.

**Figure 2:**
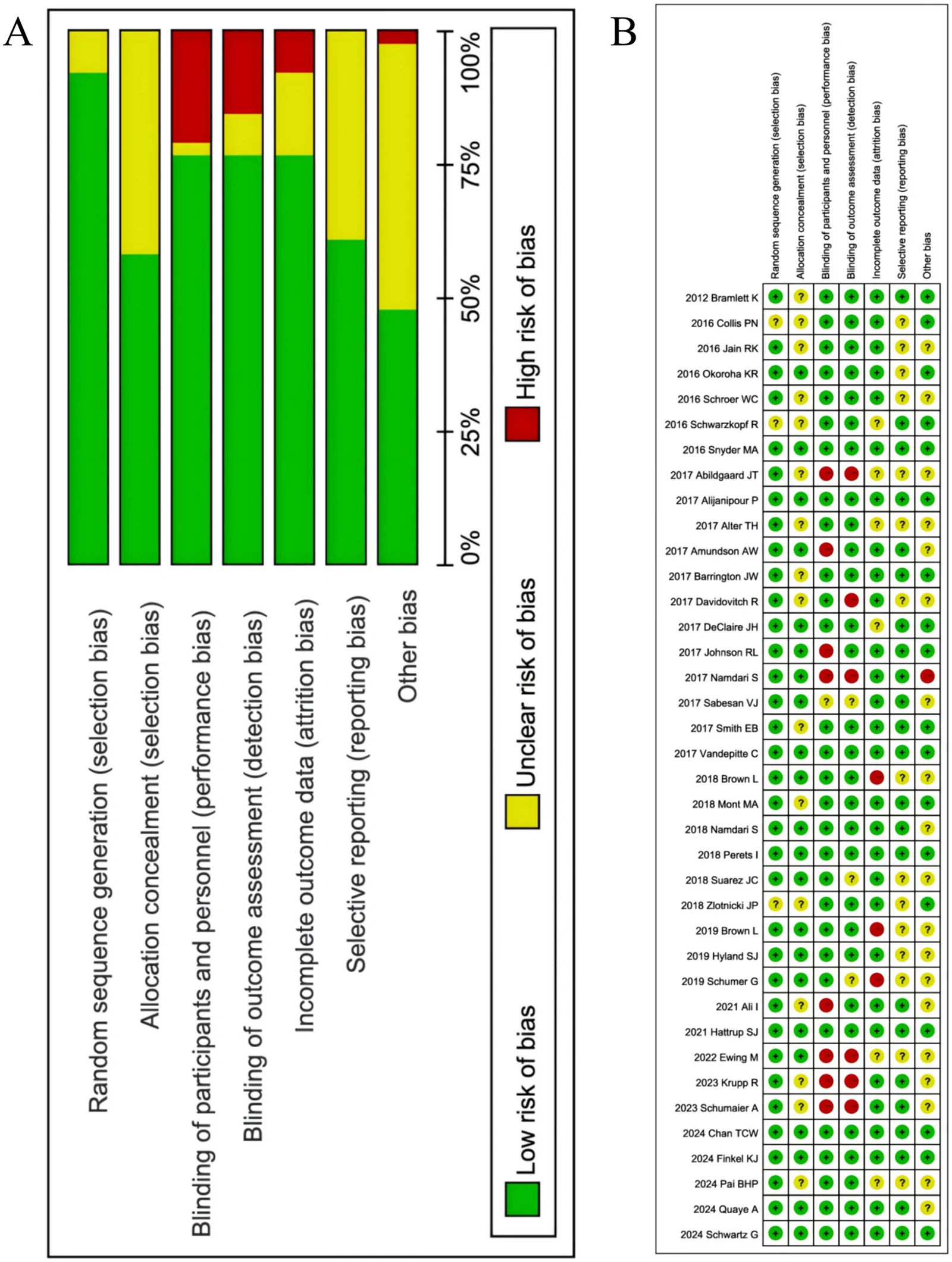
Risk of bias assessment charts for the included studies. **(A)** Green denotes low risk of bias, yellow for unclear risk of bias, and red for high risk of bias; **(B)** Green “+” denotes low risk, yellow “?” for unknown risk, and red “–” for high risk.

### 3.1 Trial characteristics

A total of 38 RCTs (16, 21, 29–64) encompassing 3,389 patients (LB: 1,689 cases, non-LB: 1,700 cases) reported on the local administration of LB for pain control in orthopedic open surgeries. The included RCTs were published from 2012 to 2025, with a significant proportion of these studies conducted in the United States. Notably, there was one study each conducted in China, Virginia, and Belgium. The open surgical procedures involved in this meta-analysis mainly included THA, TKA, lumbar fusion, total shoulder replacement (TSA) or major shoulder surgery, as well as open reduction and internal fixation for fractures of the extremities. The application of LB during the surgeries predominantly included peripheral nerve block or local infiltration, with a dosage of 133 mg or 266 mg. Furthermore, the control group was mainly treated with plain bupivacaine and ropivacaine. Trial characteristics are shown in Table 1.

**Table 1:** Summary of the characteristic of the included studies.

Author; Reference	Country	Population	Number		Age (years)		Sex (F/M)		Intervention	Control
			***E***	***C***	***E***	***C***	***E***	***C***		
Chan et al. (64)	China	DRF; Adult patients; ASA I-III; underwent open reduction and internal fixation	40	40	63 (55–69)	64 (53–70)	33/7	23/13	Supraclavicular brachial plexus block; LB (1.33%, 10 ml) in addition to PB (0.5%, 10 ml)	Supraclavicular brachial plexus block; PB (0.5%, 20 ml)
Schwartz et al. (63)	USA	Bunion deformity; undergo distal metaphyseal osteotomy procedure	81	82	52 (18–75)	47.5 (21–76)	74/7	68/14	Sciatic nerve block; LB (1.33%, 10 ml) mixed with of normal saline (0.9%, 20 ml)	Sciatic nerve block; PB (0.25%, 20 ml) mixed with normal saline (0.9%, 20 ml)
Poonam et al. (62)	USA	Scheduled to elective unilateral, primary TSA	24	24	68.00 ± 8.5	69.08 ± 6.23	13/11	14/10	Interscalene nerve block; LB (1.33%, 10 ml) with PB (0.5%, 5 ml), with a catheter left *in situ* (0.9%, saline)	Interscalene nerve block; PB (0.5%, 15 ml) with a rescue catheter left *in situ* (0.125%, PB)
Finkel et al. (61)	USA	Adult patients; underwent anatomic or reverse primary TSA with ASA I-III	45	45	72 (66.5, 77.0)	73 (66.5, 78.0)	22/23	23/22	Interscalene brachial plexus block; LB (133 mg, 10 ml) plus PB (0.5%, 10 ml)	Interscalene brachial plexus block; mixed with 0.5% PB, 5 mg dexamethasone and 5 ug epinephrine to the solution totaled 20 ml
Quaye et al. (60)	USA	Adult patients undergoing primary TKA with an ASAI–III	40	40	68.0 ± 9.0	69.1 ± 8.8	20/20	24/16	Adductor canal nerve blocks; LB (1.33%, 10 ml) mixed with PB (0.25%, 10 ml)	Adductor canal nerve blocks; PB (0.5%, 20 ml)
Krupp et al. (59)	USA	Undergoing unilateral primary or reverse TSA	21	33	66.9	67.1	7/14	18/15	Interscalene nerve block; LB (1.33%, 10 ml) mixed with PB (0.5%, 10 ml)	Interscalene nerve block; ropivacaine (0.5%, 20 ml) with an indwelling nerve block catheter delivering 0.2% ropivacaine at a continual 4 cc/h infusion
Schumaier et al. (58)	USA	Adult patients undergoing primary reverse TSA	38	38	67	68	17/21	17/21	Periarticular injection; LB (1.3%, 20 ml) diluted with normal saline (20 ml)	Interscalene nerve block; ropivacaine (0.5%, 20–30 ml)
Ewing et al. (57)	USA	Adults undergoing primary anatomic or reverse TSA	37	37	68.9 ± 8.5	70.5 ± 9.7	13/24	9/28	Interscalene nerve block; LB (133 mg, 10 ml) and PB (0.5%, 3–5 ml)	Local infiltration; ropivacaine (0.5%, 40 ml), ketorolac (30 mg, 1 ml), epinephrine (0.1 mg, 0.1 ml), and normal saline (0.9%, 58.9 ml)
Ali et al. (56)	USA	Adults aged 20–100 years; underwent primary or revision anatomic or reverse TSA	54	54	65 ± 8.7	68 ± 9.5	23/31	24/30	Local infiltration; LB (266 mg, 20 ml) diluted with 20 ml of injectable saline solution	Local infiltration; ropivacaine (0.5%, 5 mg/1 ml)
Hattrup et al. (55)	USA	Adults patients undergoing standard or reverse TSA	52	52	70.0 ± 68.4	69.2 ± 10.15	22/30	24/28	Interscalene nerve block; LB (133 mg, 10 ml) with 7.5 ml of 0.5% and 7.5 ml of 0.25% plain bupivacaine	Interscalene nerve block; PB (0.5%, 25 ml)
Hyland et al. (21)	USA	English-speaking patients; 18 years of age or older who planned to receive a unilateral TKA	30	29	65.0 ± 8.3	61.2 ± 8.9	16/14	16/13	Periarticular injection; LB (266 mg, 60 ml)	Periarticular injection; ropivacaine (0.2%, 20 ml), morphine (10 mg), ketorolac (30 mg), and methylprednisolone (40 mg).
Schumer et al. (53)	Virginia	Adult patients requiring TKA for the management of primary steoarthritis of the knee	66	64	–	–	–	–	Local infiltration; LB (266 mg, 20 ml)	Local infiltration; PB (0.5, 60 ml)
Brown et al. (52, 54)	USA	Degenerative spondylosis; Adult patients undergoing a primary, 1- or 2-level elective open PLIF	33	26	59.8 ± 12.22	63.2 ± 10.70	12/21	17/9	Local infiltration; LB (266 mg, 60 ml)	Local infiltration; sterile saline (0.9%, 60 ml)
Mont et al. (48)	USA	Undergo primary, unilateral, ricompartmental TKA under spinal anesthesia	70	69	66 ± 8.61	66 ± 7.21	43/27	39/30	Local infiltration; LB (266 mg, 20 ml) admixed with PB (0.5%, 20 ml)	Local infiltration; PB (0.5%, 20 ml)
Perets yet al. (51)	USA	Undergo primary THA under general anesthesia	50	57	61.9	62.4	29/21	31/26	Periarticular injection; 20 ml LB, 40 ml 0.25% PB with epinephrine, and 20 ml of normal saline	Periarticular injection; PB (0.25%, 60 ml) with epinephrine
Namdari et al. (50)	USA	Undergoing primary total or reverse TSA for osteoarthritis or cuff tear arthropathy	39	39	68.6 ± 10.00	71.2 ± 8.6	20/19	15/24	Interscalene brachial plexus block; LB (266 mg, 20 ml)	Interscalene brachial plexus block; ropivacaine (0.5%, 15 ml)
Suarez et al. (49)	USA	Patients scheduled for a primary TKA; 18–85 years of age	52	52	68.1 ± 8.20	67.3 ± 7.40	33/19	26/26	Periarticular injection; LB (266 mg, 20 ml), PB (0.25%, 30 ml), normal saline (0.9%, 50 ml)	Periarticular injection cocktail: PB (0.25%, 30 ml), lidocaine with epinephrine (1%, 15 ml), 10 mg morphine, 60 mg ketorolac, normal saline (0.9%, 50 ml)
Zlotnicki et al. (47)	USA	Cemented primary TKA; receive regional anesthetic with intrathecal morphine injection	38	40	63.2 ± 7.2	64.3 ± 8.8	19/19	26/14	Periarticular injection; LB (266 mg, 20 ml) mixed with 70 ml normal saline	Periarticular injection; PB (0.5%, 20 ml) mixed with 70 ml normal saline
Smith et al. (36)	USA	18 years of age or older Scheduled to be treated with primary unilateral TKA	104	96	66 (41–90)	66 (47–89)	50/54	68/28	Periarticular injection; LB (266 mg 20 ml) combined with a dummy, inactivated pain pump loaded with saline solution	Active pain pump bupivacaine hydrochloride
Alijanipour et al. (37)	USA	Adult patients 18-80 years old and underwent unilateral primary TKA for osteoarthritis	59	59	64.3	64.9	30/29	32/27	Periarticular injection; LB (266 mg, 20 ml) with normal saline (40 ml) and epinephrine (0.5 mg, 0.5 ml)	Periarticular injection; 20 ml (50 mg) of free bupivacaine solution (Marcaine 0.25% with epinephrine 1:200,000) mised with 40 ml of normal saline
DeClaire et al. (38)	USA	Unilateral TKA; 18 years of age or older; Diagnosis of degenerative osteoarthritis of the knee	47	49	69.7 ± 8.6	67.7 ± 9	27/20	28/21	Periarticular injection; LB (1.3%, 20 ml), bupivacaine, ketorolac, morphine, and epinephrine	Periarticular injection; standard “pain cocktail” injection: ropivacaine, ketorolac, morphine, and epinephrine
Barrington et al. (39)	USA	Undergoing primary unilateral TKA; At least 18 years of age	40	38	69 ± 9	69 ± 9	29/11	26/12	Periarticular injection; LB (1.3%, 20 ml), PB (0.5%, 25 ml), 30 mg ketorolac, and 1 mg of 1:1,000 epinephrine	Periarticular injection; ropivacaine (0.5%, 50 ml), 30 mg ketorolac, and 1 mg of 1:1,000 epinephrine
Amundson et al. (40)	USA	Patients older than or equal to 18 years; ASA I to III; Undergoing an elective, unilateral primary TKA	52	55	67 ± 8	68 ± 8	27/25	34/21	Periarticular injection; LB (266 mg, 20 ml)	Periarticular injection; weight-based solution with ropivacaine
Davidovitch et al. (41)	USA	Isolated acute rotational ankle fracture; Over 18 years of age; sustained an acute ankle fracture requiring operative fixation	37	39	41.9	42.6	19/18	20/19	Local infiltration; 40 ml solution consisting of 1.3% LB and sterile saline	Local infiltration; 40 ml of normal saline
Vandepitte et al. (42)	Belgium	ASA I-III, 18 years or older who were scheduled for elective major shoulder surgery (rotator cuff repair or TSA)	26	24	61 ± 11	57 ± 12	13/13	14/10	Interscalene brachial plexus block; LB (133 mg, 10 ml) and PB (0.25%, 5 ml)	Interscalene brachial plexus block; PB (0.25%, 15 ml)
Sabesan et al. (43)	USA	Undergoing primary TSA; ASA I-III, patients aged 18 years or older	34	36	63	65	9/25	17/19	Local infiltration; LB (266 mg, 20 ml) and normal saline (0.9%, 60 ml)	Single-dose interscalene nerve block with 0.5% SB (0.5%, 20 ml), ACTion Block Pain Pump with 0.125% bupivacaine at a rate of 6 ml/h postoperatively.
Namdari et al. (44)	USA	Undergoing primary TSA for osteoarthritis or reverse arthroplasty for cuff tear arthropathy	78	78	68.4 ± 8.2	70.9 ± 9.3	38/40	47/31	Local infiltration; LB (266 mg, 20 ml)	Local infiltration; ropivacaine (0.5%, 30 ml)
Abildgaard et al. (45)	USA	Patients older than 18 years; candidates for a TSA with either an anatomic or reverse prosthesis	37	46	–	–	16/21	32/14	Local infiltration; LB (266 mg) mixed in 40 ml of saline	Nerve block; 0.5% ropivacaine with catheters set to administer 8 ml/h.
Johnson et al. (46)	USA	Adult patients; ASA I-III; undergoing elective primary THA	54	54	61.7 ± 11.7	59.4 ± 1.3	28/26	27/27	Periarticular injection; LB 266 mg, ketorolac 30 mg, bupivacaine 125 mg, and epinephrine 125 mg	Periarticular injection; ropivacaine, epinephrine, and ketorolac
Alter et al. (16)	USA	Patients age greater than 18 years; an indication for outpatient DRF repair surgery using volar locked plate fixation	20	21	57 ± 15	63 ± 15	16/4	17/4	Local infiltration; 10 ml 0.5% Marcaine; 10 ml LB into the same site upon completion of the surgery and wound closure	Local infiltration; 20 ml 0.5% bupivacaine without epinephrine into the incision and surgical site before incision
Okoroha et al. (35)	USA	Patients older than 18 years undergoing either primary anatomic or reverse TSA	26	31	69.4	67.1	14/12	15/16	Local infiltration; LB (266 mg, 20 ml) mixed in 20 ml of sterile saline	Interscalene brachial plexus block; ropivacaine (0.5%, 40 ml)
Collis et al. (34)	USA	Adult patients underwent unilateral, primary TKA with the principle diagnosis of primary osteoarthritis.	54	51	63.7 ± 8.8	63.5 ± 10.1	29/25	37/14	Periarticular injection; LB in a 20 ml vial	Periarticular injection; 49.25 ml ropivacaine, 0.5 mg epinephrine (1 mg/ml, 0.5 ml), 30 mg ketorolac, 0.8 ml clonidine, diluted with 48.45 ml of saline
Schwarzkopf et al. (33)	USA	Primary TKA with a history of chronic opioid use	20	18	63 (57–66)	59 (53–65)	9/11	12/6	Periarticular injection; LB 20 mL mixed with 60 mL saline, and 20 mL 0.25% Marcaine injected with a different syringe	Periarticular injection; ropivacaine (0.5%, 49.25 mL), clonidine (100 mg/mL, 0.8 mL), Toradol (30 mg/mL, 1 mL), Epinepherine (1 mg/mL, 0.5 mL), and 48 mL saline
Snyder et al. (32)	USA	At least 18 years of age; received a TKA for degenerative joint disease, due to failing all other non-operative treatments to control pain	35	35	67.31 ± 10.27	65.57 ± 7.89	13/22	20/15	Periarticular injection; 266 mg LB suspension reconstituted to 100 mL with 0.9% normal saline	Periarticular injection; ketorolac 30 mg, morphine PF 5 mg, epinephrine 0.6 mg, ropivicaine 400 mg, quantity sufficient to 100 ml with 0.9% normal saline
Jain et al. (31)	USA	Scheduled to have a unilateral TKA	63	62	68.3 ± 8.4	67.5 ± 8.3	44/19	45/17	Periarticular injection; LB (266 mg, 20 ml)	Periarticular injection; 30 ml of 0.25% bupivacaine with 1:200,000 epinephrine and 10 mg of morphine throughout the joint
Schroer et al. (30)	USA	Undergoing unilateral primary TKA	58	53	67 ± 8.8	68.6 ± 9.2	34/24	32/21	Periarticular injection; LB (266 mg, 20 ml) combined with SB (75 mg, 30 ml)	Periarticular injection; PB (150 mg, 60 ml)
Bramlett et al. (29)	USA	Adults 18–75 years of age with ASA 1–3; scheduled to undergo primary unilateral TKA under general anesthesia	25	34	61.1 ± 8.7	62.2 ± 7.2	12/13	23/11	Local infiltration; 266 mg LB diluted with 0.9% saline to a total volume of 60 ml	Local infiltration; 150 mg PB diluted with preservative-free 0.9% saline to a total volume of 60 ml

*E*, experimental group; C, control group; F, female; M, male; DRF, distal radial fracture; ASA, American Society of Anesthesiologists Physical Status Classification; LB, liposomal bupivacaine; PB, plain bupivacaine; TSA, total shoulder arthoplasty; TKA, total knee arthoplasty; PLIF, posterior lumbar interbody fusion; THA, total hip arthoplasty.

### 3.2 Meta-analysis results of postoperative pain intensity scores

#### 3.2.1 Pain intensity scores on POD0

Nine studies (32, 35, 36, 39, 43, 44, 57, 58, 64) reported pain intensity scores on postoperative day 0 (POD0). Significant heterogeneity were observed among the studies (*I*^2^ = 82.7%, *P* < 0.001), and the data were therefore pooled using a random-effects model. The meta-analysis results demonstrated no statistically significant difference in pain intensity scores on POD0 between the two groups [SMD = 0.09, 95% CI (−0.25 to 0.42); Figure 3].

**Figure 3:**
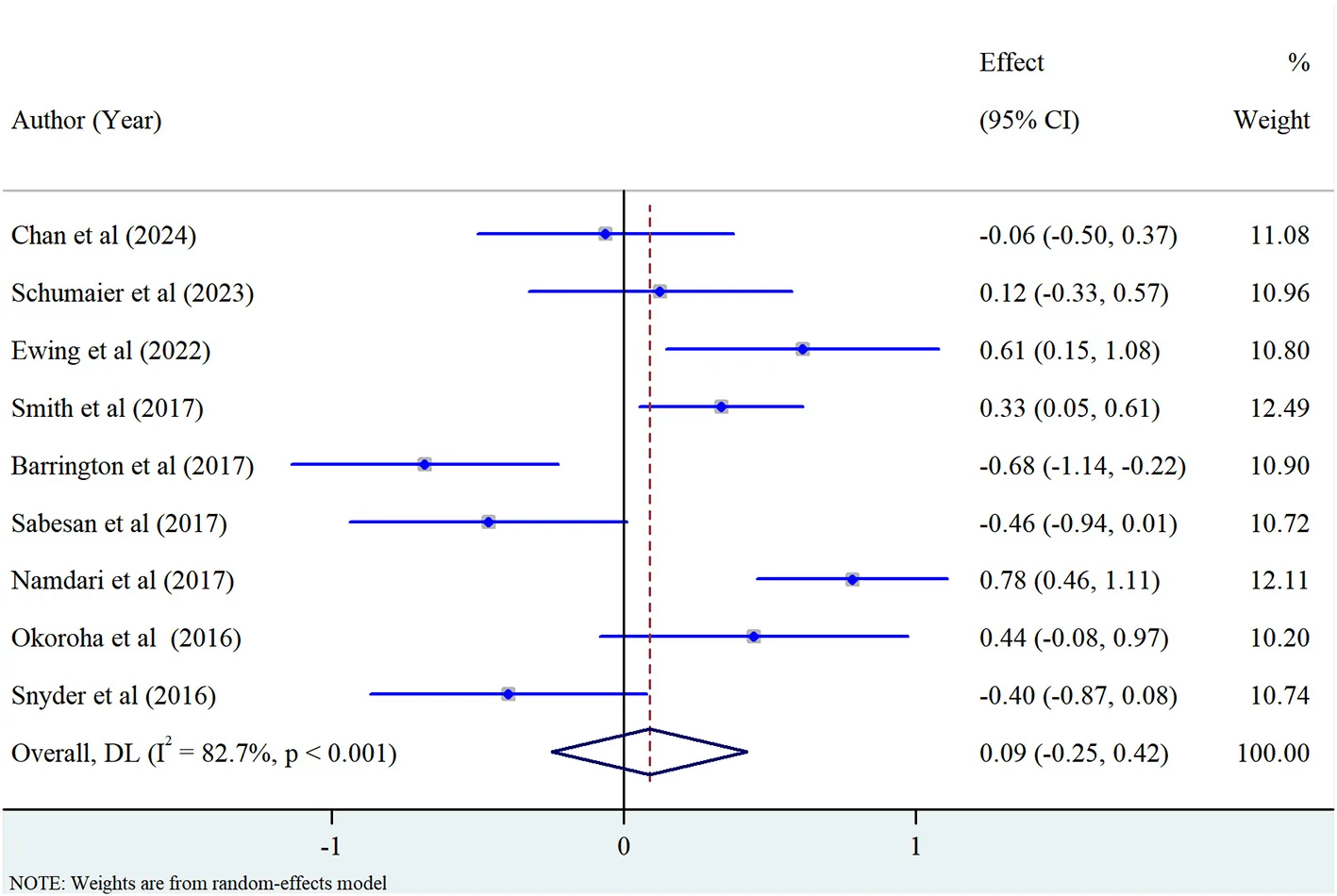
Forest plot of the meta-analytic estimate for pain intensity scores on postoperative day 0 (POD0).

#### 3.2.2 Pain intensity scores on POD1

Eighteen studies (21, 29–32, 35, 39, 43, 44, 52, 53, 55–59, 63, 64) reported pain intensity scores on postoperative day 1 (POD1). Significant heterogeneity was observed among the studies (*I*^2^ = 68.4%, *P* < 0.001), and a random-effects model was used to pool the data. The results of the meta-analysis indicated a marginal significant difference in pain intensity scores on POD1 [SMD = −0.17, 95% CI (−0.35 to 0.00); Figure 4A].

**Figure 4:**
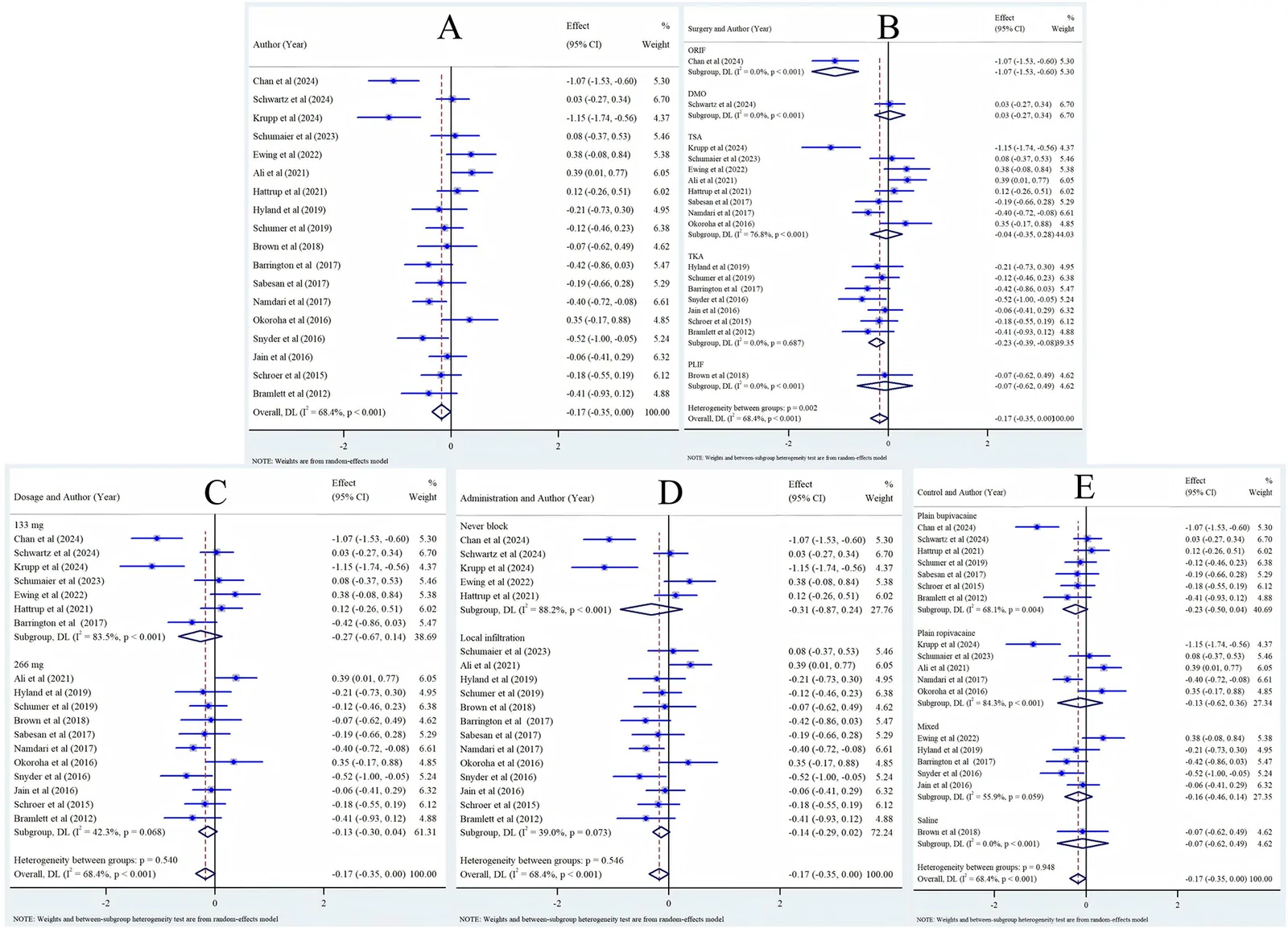
Forest plot of the meta-analytic estimate for pain intensity scores on postoperative day 1 (POD1) **(A)**, along with subgroup analyses stratified by surgical procedure type **(B)**, liposomal bupivacaine dosage **(C)**, administration technique **(D)**, and interventions in the control group **(E)**.

Subgroup analysis revealed that patients undergoing TKA [SMD = −0.23, 95% CI (−0.39 to −0.08); Figure 4B] and open reduction and internal fixation for limb fractures [SMD = −1.07, 95% CI (−1.53 to −0.60); Figure 4B] in the LB group experienced a statistically significant reduction in pain intensity scores on POD1. However, no significant differences were observed in subgroups based on other types of surgery (Figure 4B), LB dosages (Figure 4C), administration techniques (Figure 4D), and control group interventions (Figure 4E).

#### 3.2.3 Pain intensity scores on POD2

Fourteen studies (29, 30, 32, 35, 39, 43, 48, 52, 55, 57–59, 63, 64) reported pain intensity scores on postoperative day 2 (POD2). Significant heterogeneity was detected among the studies (*I*^2^ = 75.6%, *P* < 0.001), and the data were pooled using a random-effects model. The results showed that pain intensity scores on POD2 were significantly lower in the LB group compared to the control group [SMD = −0.25, 95% CI (−0.49 to −0.02); Figure 5A].

**Figure 5:**
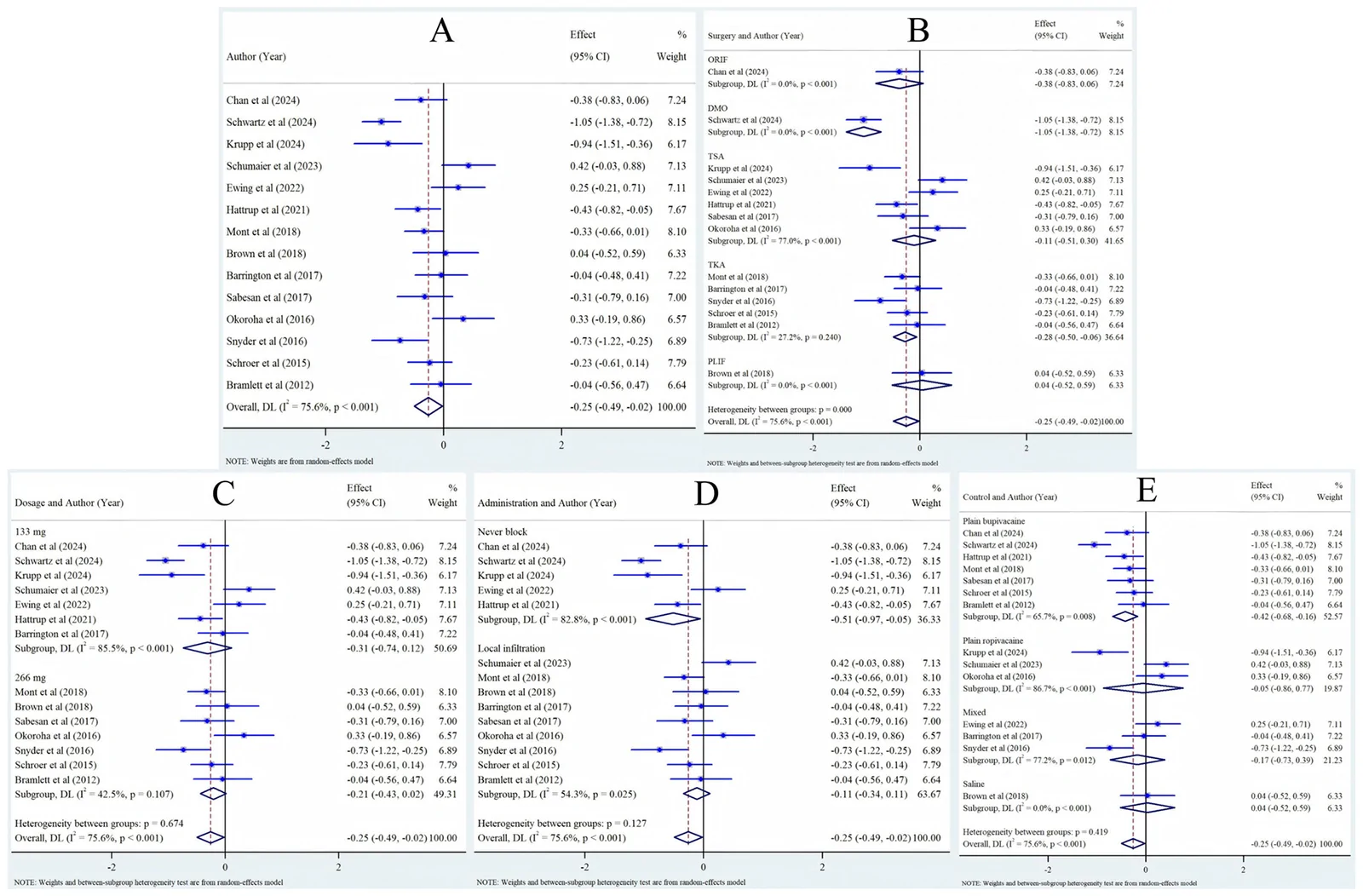
Forest plot of the meta-analytic estimate for pain intensity scores on postoperative day 1 (POD2) **(A)**, along with subgroup analyses stratified by surgical procedure type **(B)**, liposomal bupivacaine dosage **(C)**, administration technique **(D)**, and interventions in the control group **(E)**.

Subgroup analysis revealed that the use of LB was associated with lower pain intensity scores on POD2 in patients undergoing TKA [SMD = −0.28, 95% CI (−0.50 to −0.06); Figure 5B] and distal metaphyseal osteotomy (DMO) procedures [SMD = −1.05, 95% CI (−1.38 to −0.72); Figure 5B]. However, no significant difference was detected in LB dosage (133 mg versus 266 mg) between the groups (Figure 5C). Furthermore, statistically significant reductions in postoperative pain scores were observed when LB was administered via nerve block [SMD = −0.51, 95% CI (−0.97 to −0.05); Figure 5D], or when plain bupivacaine was used as the intervention in the control group [SMD = −0.42, 95% CI (−0.68 to −0.16); Figure 5E].

#### 3.2.4 Pain intensity scores on POD3

Eleven studies (29, 30, 32, 35, 39, 52, 55, 57, 59, 63, 64) reported pain intensity scores on postoperative day 3 (POD3). Significant heterogeneity was observed among the studies (*I*^2^ = 75.3%, *P* < 0.001), and the data was pooled using a random-effects model. The results of the meta-analysis demonstrated no statistically significant difference in pain intensity scores on POD3 between the two groups [SMD = −0.22, 95% CI (−0.49 to 0.05); Figure 6A].

**Figure 6:**
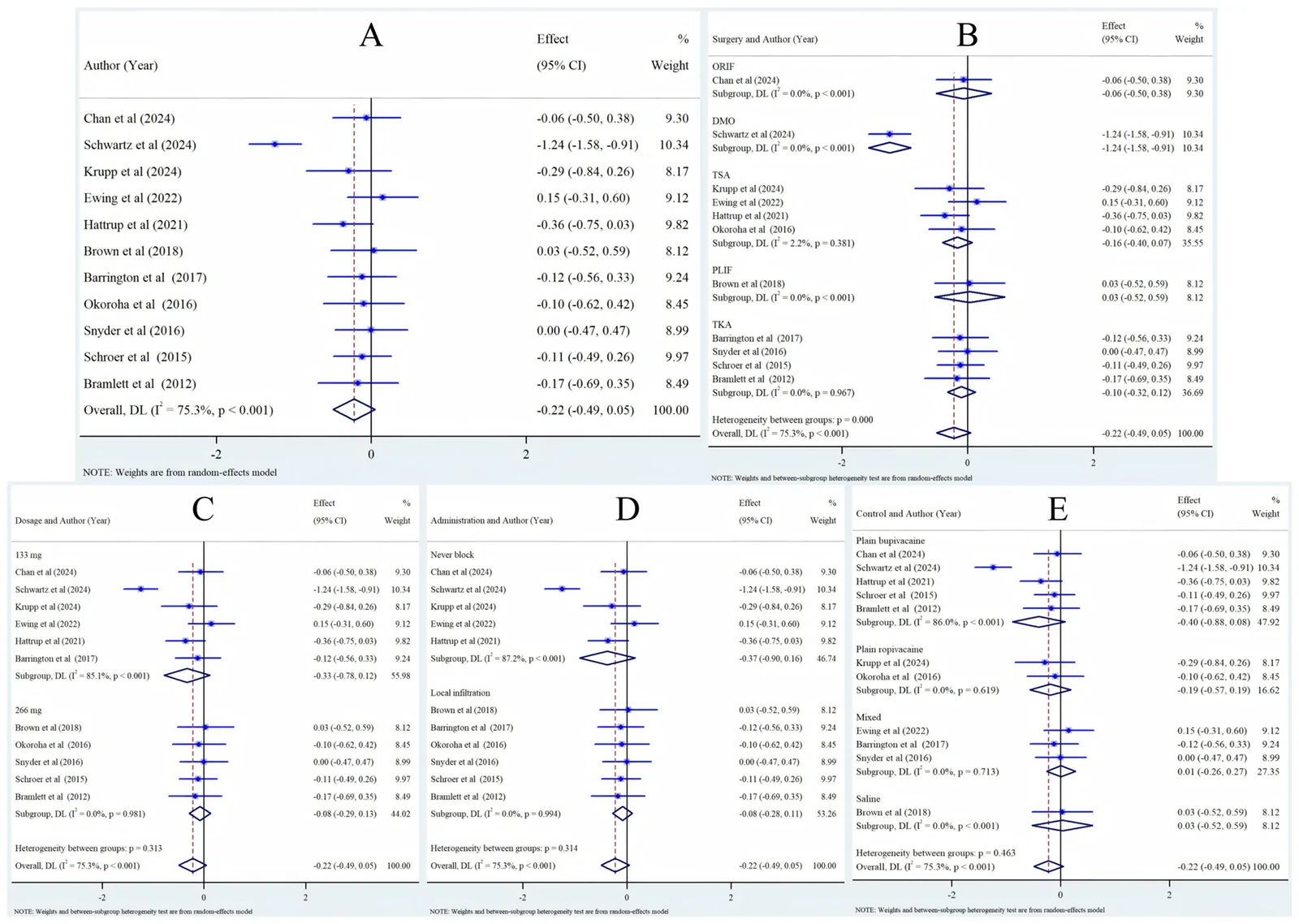
Forest plot of the meta-analytic estimate for pain intensity scores on postoperative day 1 (POD3) **(A)**, along with subgroup analyses stratified by surgical procedure type **(B)**, liposomal bupivacaine dosage **(C)**, administration technique **(D)**, and interventions in the control group **(E)**.

However, the subgroup analysis revealed that, with the exception of patients with bunion deformities patients undergoing DMO [SMD = −1.24, 95% CI (−1.58 to −0.91); Figure 6B], no statistically significant differences in pain intensity scores on POD3 were observed with respect to other types of surgery (Figure 6B), LB dosages (Figure 6C), administration techniques (Figure 6D), or the interventions in the control group (Figure 6E).

### 3.3 Meta-analysis results of opioid consumption in morphine equivalents

#### 3.3.1 Total opioid consumption

Fourteen studies (21, 30, 31, 36, 39, 42, 44, 48, 51–53, 55, 57, 63) reported total opioid consumption among the groups. Significant heterogeneity was observed among the studies (*I*^2^ = 90.9%, *P* < 0.001), and a random-effects model was employed to pool the data. The meta-analysis results indicated no significant difference in postoperative total opioid consumption between the two groups [SMD = −0.27, 95% CI (−0.61 to 0.08); Figure 7A].

**Figure 7:**
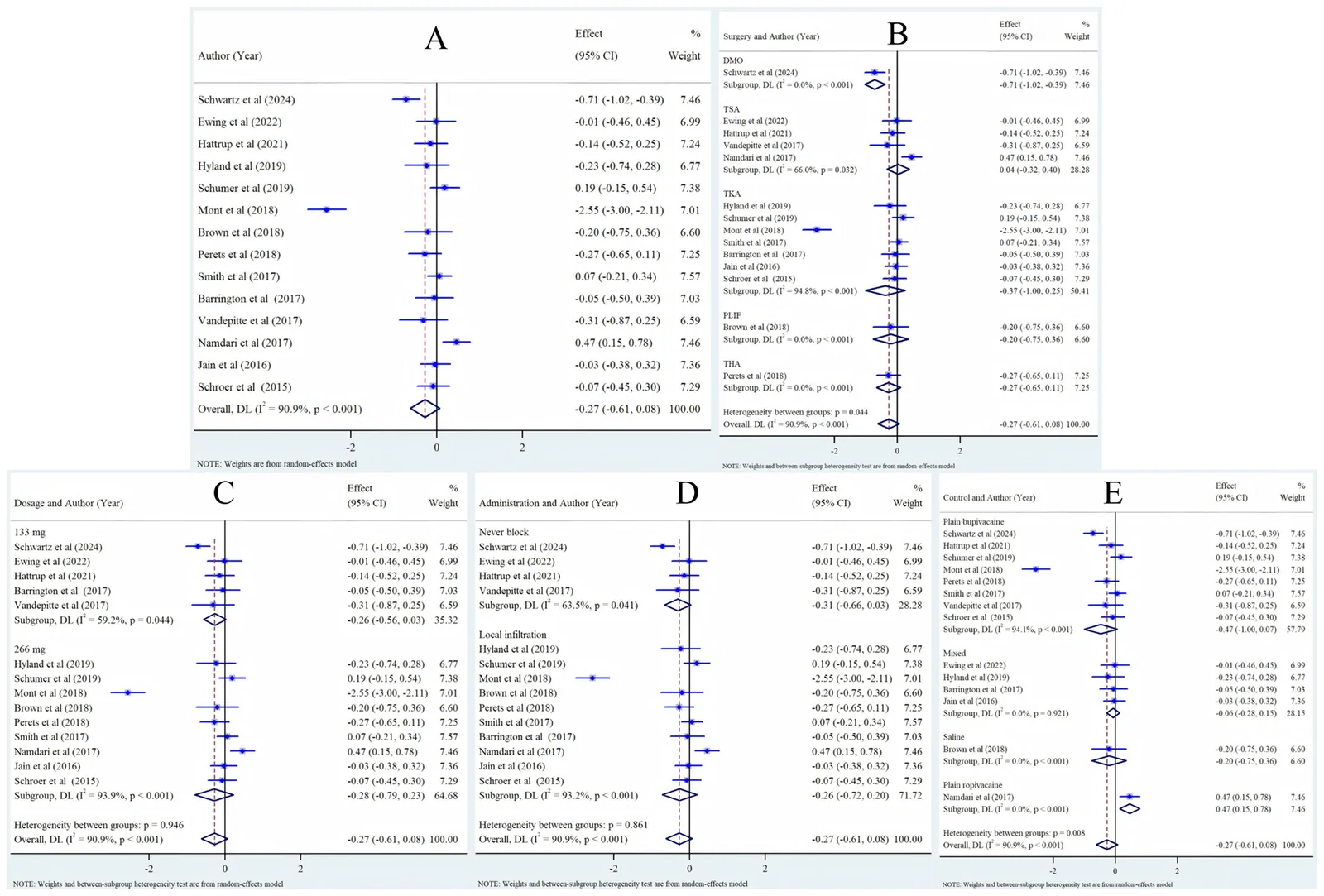
Forest plot of the meta-analytic estimate for total opioid consumption **(A)**, along with subgroup analyses stratified by surgical procedure type **(B)**, liposomal bupivacaine dosage **(C)**, administration technique **(D)**, and interventions in the control group **(E)**.

Subgroup analysis revealed that patients with bunion deformities undergoing DMO in the LB group experienced a statistically significant reduction in total opioid consumption [SMD = −0.71, 95% CI (−1.02 to −0.39); Figure 7B]. Furthermore, the LB group demonstrated a statistically significant improvement in total opioid consumption compared to the plain ropivacaine group [SMD = 0.47, 95% CI (0.15 to 0.78); Figure 7E]. However, no significant differences were observed in subgroups stratified by other types of surgery (Figure 7B), LB dosages (Figure 7C), administration techniques (Figure 7D), and other control group interventions (Figure 7E).

#### 3.3.2 Opioid consumption on POD1

Eleven studies (32, 35, 42, 44, 50, 51, 56–59, 63) reported opioid consumption on POD1 among the groups. Low heterogeneity was observed among the studies (*I*^2^ = 49.0%, *P* = 0.033), and a fixed-effects model was employed to pool the data. The meta-analysis results indicated no statistically significant difference in opioid consumption on POD1 between the two groups [SMD = 0.10, 95% CI (−0.03 to 0.22); Figure 8].

**Figure 8:**
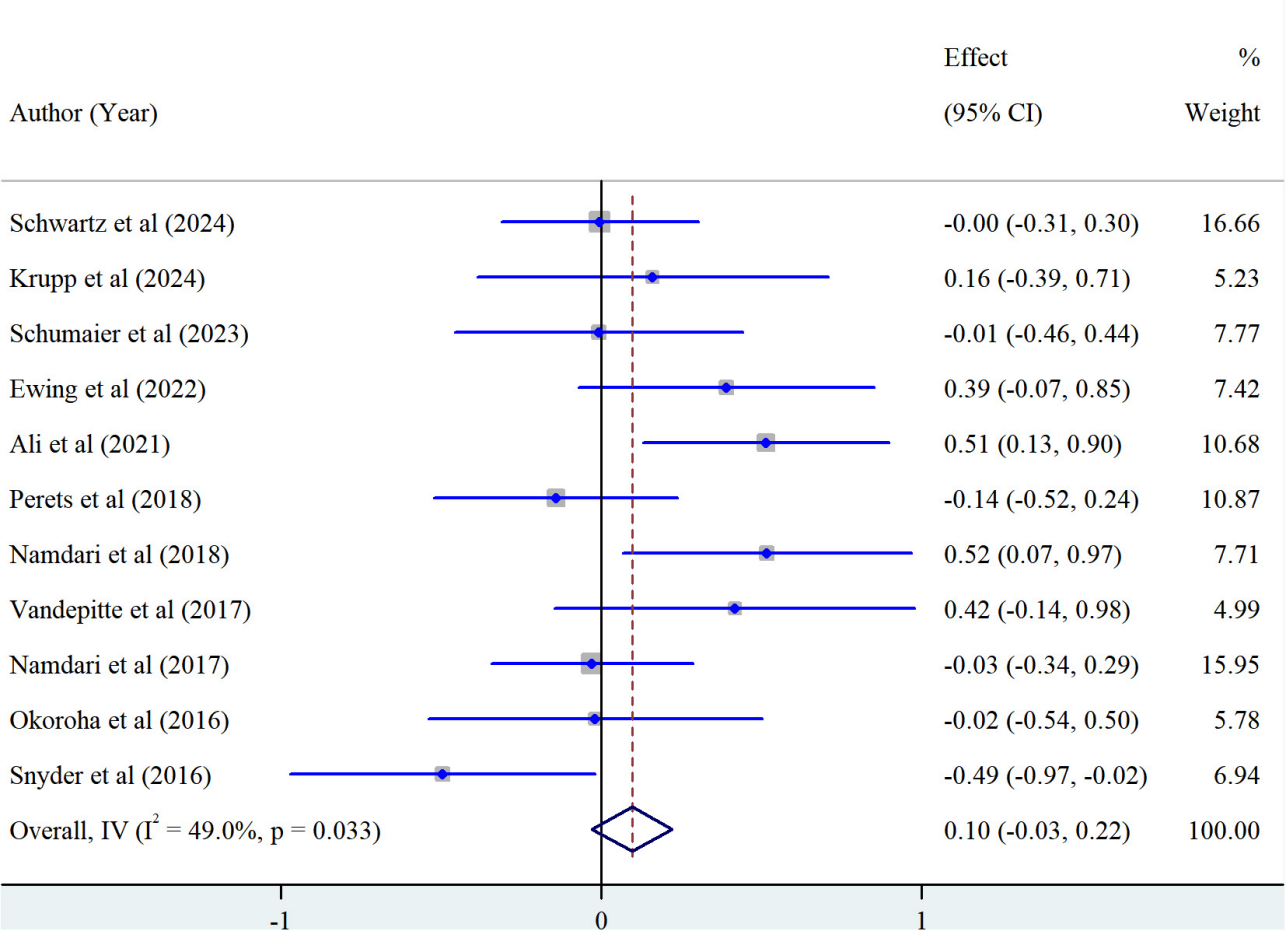
Forest plot of the meta-analytic estimate for opioid consumption on POD1 **(A)**, along with subgroup analyses stratified by surgical procedure type **(B)**, liposomal bupivacaine dosage **(C)**, administration technique **(D)**, and interventions in the control group **(E)**.

#### 3.3.3 Opioid consumption on POD2

Ten studies (32, 35, 42, 48, 51, 56–59, 63) reported opioid consumption on POD2 across the groups. Significant heterogeneity was observed among the studies (*I*^2^ = 94.2%, *P* < 0.001), and the data were pooled using a random-effects model. The results of the meta-analysis revealed no statistically significant difference in opioid consumption between the groups on POD2 [SMD = −0.25, 95% CI (−0.84 to 0.33); Figure 9A].

**Figure 9:**
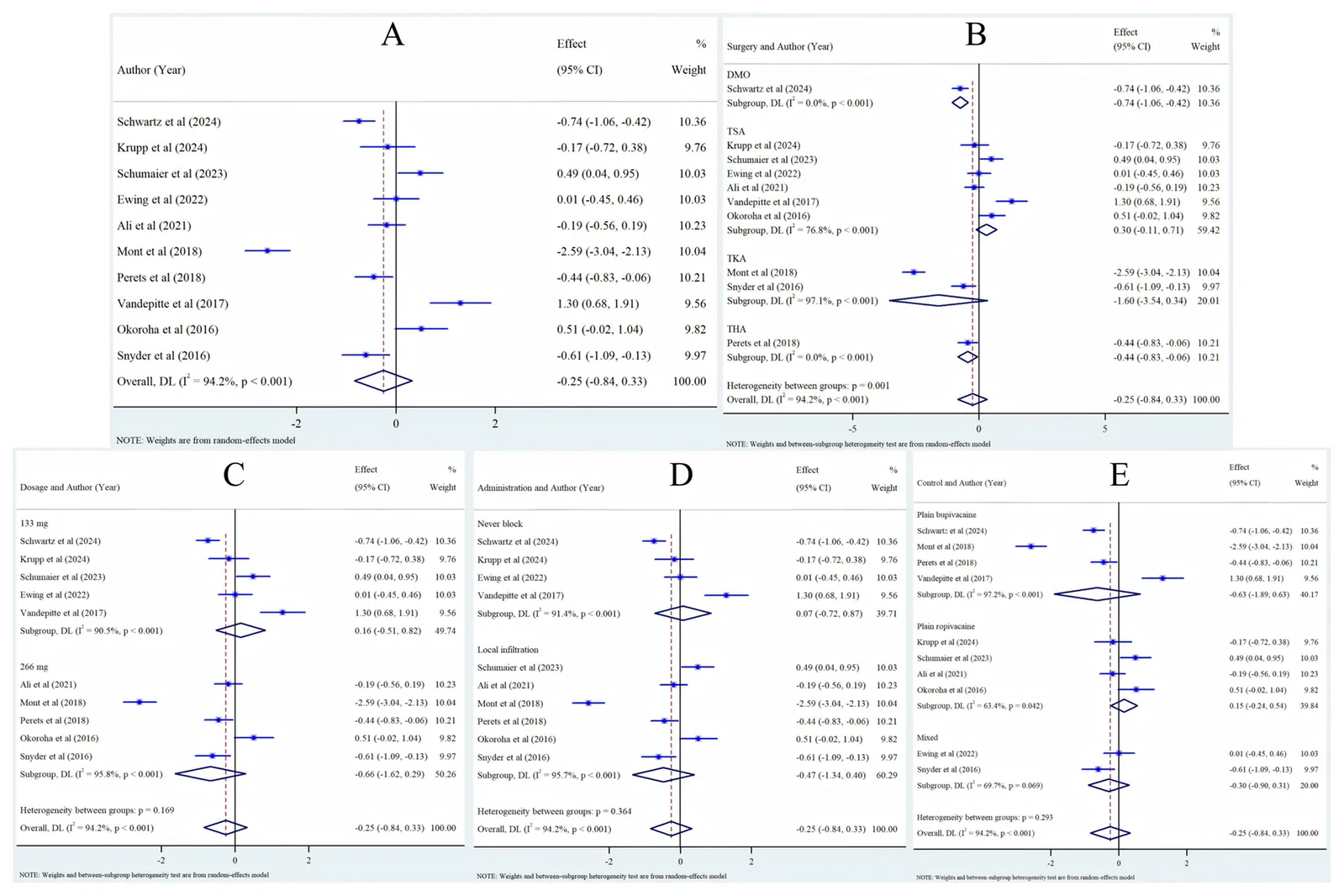
Forest plot of the meta-analytic estimate for opioid consumption on POD2 **(A)**, along with subgroup analyses stratified by surgical procedure type **(B)**, liposomal bupivacaine dosage **(C)**, administration technique **(D)**, and interventions in the control group **(E)**.

Subgroup analysis revealed that patients undergoing DMO [SMD = −0.74, 95% CI (−1.06 to −0.42); Figure 9B] and THA [SMD = −0.44, 95% CI (−0.83 to −0.66); Figure 9B] in the LB group experienced a statistically significant reduction in opioid consumption on POD2. However, no significant differences were observed in subgroups categorized by other types of surgery (Figure 9B), LB dosages (Figure 9C), administration techniques (Figure 9D), and control group interventions (Figure 9E).

#### 3.3.4 Opioid consumption on POD3

Ten studies (32, 35, 42, 48, 51, 55–57, 59, 63) reported opioid consumption on POD3 among the groups. Significant heterogeneity was observed among the studies (*I*^2^ = 93.0%, *P* < 0.001); therefore, a random-effects model was employed to pool the data. The results of meta-analysis indicated no significant difference in opioid consumption between the two groups on POD3 [SMD = −0.40, 95% CI (−0.92 to 0.12); Figure 10A].

**Figure 10:**
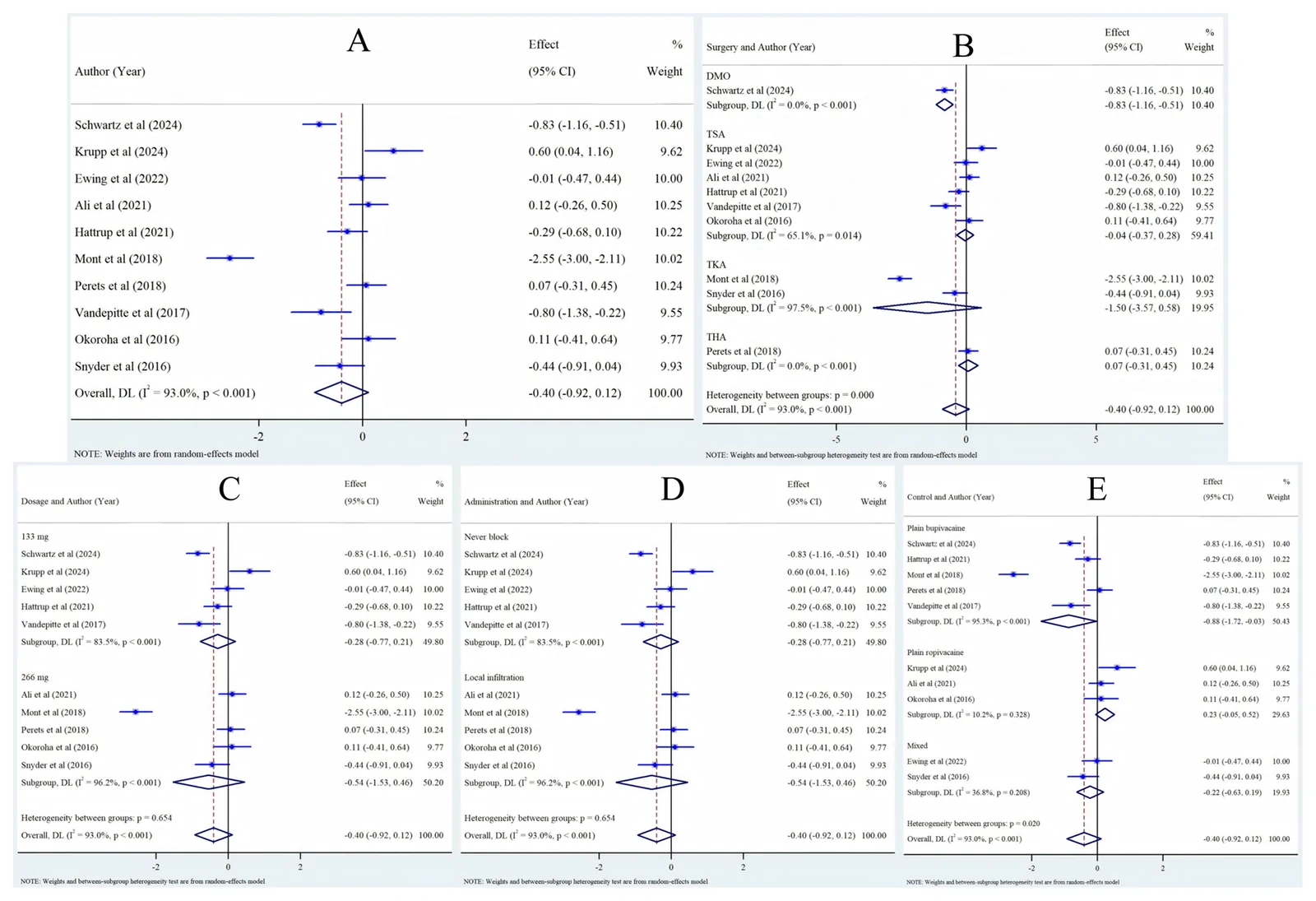
Forest plot of the meta-analytic estimate for opioid consumption on POD3 **(A)**, along with subgroup analyses stratified by surgical procedure type **(B)**, liposomal bupivacaine dosage **(C)**, administration technique **(D)**, and interventions in the control group **(E)**.

In subgroup analysis, the use of LB was associated with a statistically significant decrease in opioid consumption on POD3 among patients with bunion deformities undergoing DMO [SMD = −0.83, 95% CI (−1.16 to −0.51); Figure 10B]. Furthermore, LB significantly reduced opioid consumption on POD3 compared with plain bupivacaine [SMD = −0.88, 95% CI (−1.72 to −0.03); Figure 10E]. However, no significant differences were observed in subgroups stratified by other types of surgery (Figure 10B), LB dosages (Figure 10C), administration techniques (Figure 10D), and other control group interventions (Figure 10E).

### 3.4 Meta-analysis results of patient satisfaction, length of hospital stay

Seven studies (32, 36, 42, 43, 55, 56, 58) reported patient satisfaction following LB administration and other control interventions, with significant heterogeneity observed among the studies (*I*^2^ = 92.7%, *P* < 0.001), and the data were pooled using a random-effects model. The results of the meta-analysis indicated no significant difference in patient satisfaction among the groups [SMD = −0.48, 95% CI (−1.09 to 0.13); Figure 11A]. Moreover, no statistically significant difference was found in the length of hospital stay between the LB group and the control group [SMD = 0.02, 95% CI (−0.10 to 0.13); Figure 11B].

**Figure 11:**
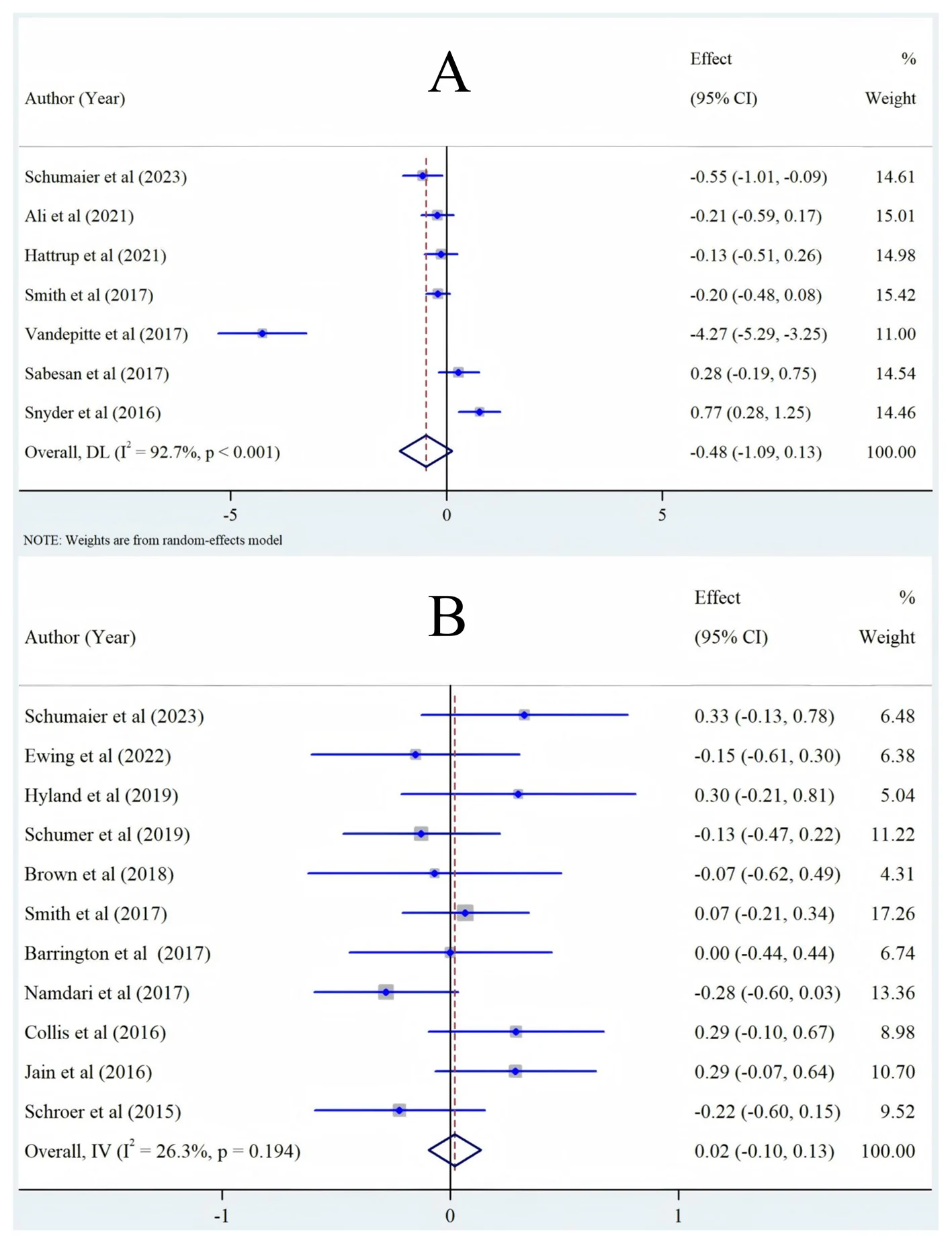
Forest plot of the meta-analytic estimate for patient satisfaction **(A)** and the length of hospital stay **(B)**.

### 3.5 Meta-analysis results of treatment-emergent adverse events

Twenty-six studies (16, 21, 29, 33, 35–46, 48, 49, 51, 56–58, 61–64) reported treatment-emergent adverse events (TEAEs), with no significant heterogeneity observed among the studies (*I*^2^ = 0.1%, *P* = 0.456). A fixed-effect model was used for the data pooling. The results of this meta-analysis demonstrated that the incidence of TEAEs in the LB group was not significantly higher than in the control group, with no significant difference [risk ratio (RR) = 1.05, 95% CI (0.91 to 1.20); Figure 12]. Furthermore, we performed the subgroup analysis based on the types of opioid-related adverse events. The results showed that no significant differences in the incidence of nausea/vomiting [RR = 0.98, 95% CI (0.80 to 1.20); Figure 12], constipation [RR = 0.91, 95% CI (0.61 to 1.35); Figure 12], pruritus [*R* = 1.30, 95% CI (0.78 to 2.16); Figure 12], and dizziness [RR = 0.71, 95% CI (0.38 to 1.33); Figure 12].

**Figure 12:**
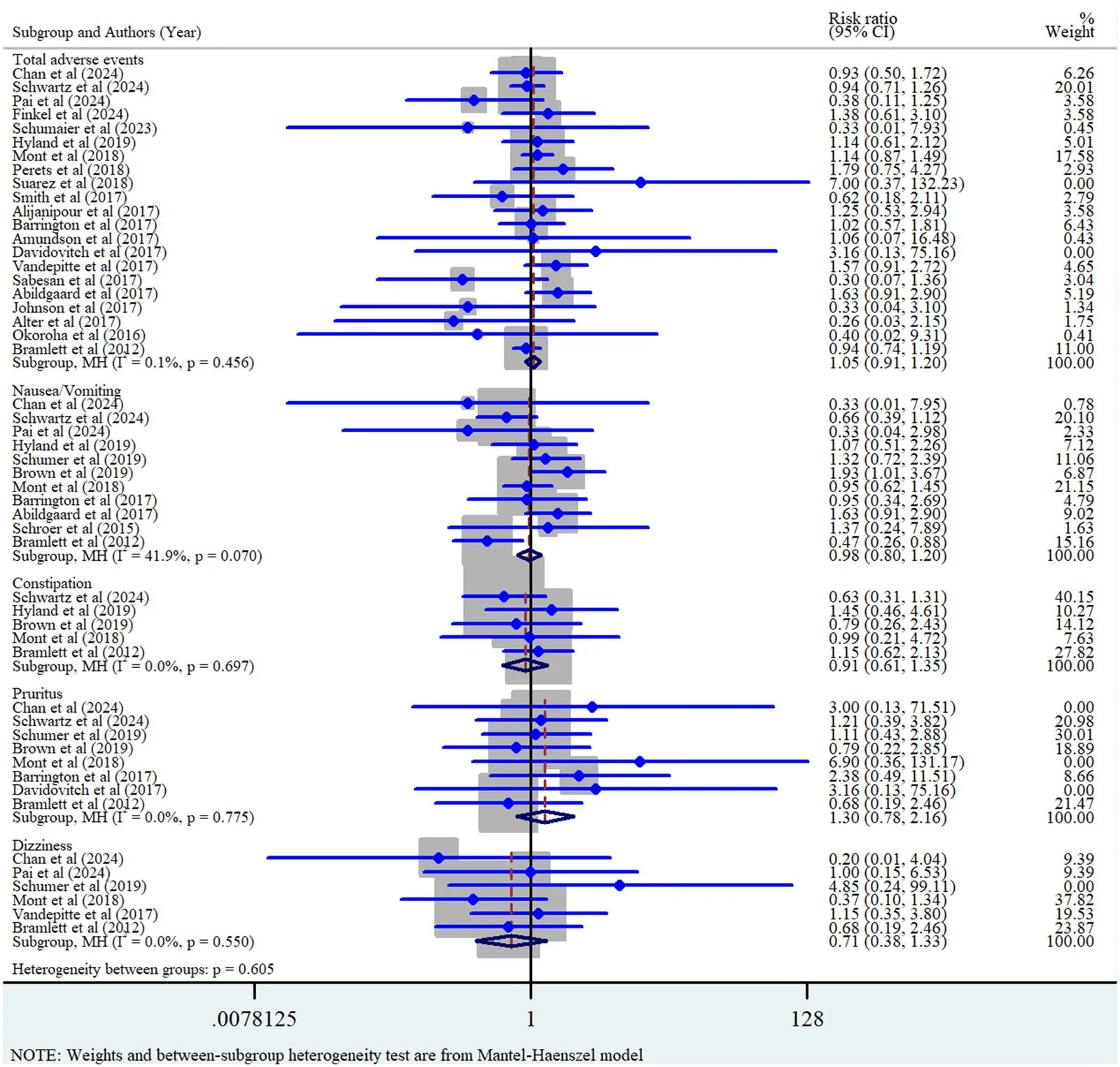
Forest plot of the meta-analytic estimate for adverse events.

### 3.6 Sensitively analysis, meta-regression, and publication bias

We conducted a sensitivity analysis (using the stepwise exclusion method, influence analyses) for outcome indicators that included more than 10 studies. The results of the sensitivity analysis demonstrated that excluding any single study did not significantly alter the pooled effect size of the remaining studies, which remained consistent with the previous meta-analysis results (Table 2). This indicates that these outcome indicators have good robustness and strong evidence strength.

**Table 2:** Sensitively analysis for the outcomes with more than 10 studies.

Outcomes	No. of included study	Effect size (SMD/RR, 95%CI) (meta-analysis)	Effect size (SMD/RR, 95%CI) (sensitively analysis)
Pain intensity scores (POD1)	18	−0.17 (−0.35 to 0.00)	−0.17 (−0.35 to 0.004)
Pain intensity scores (POD2)	14	−0.25 (−0.49 to −0.02)	−0.25 (−0.49 to −0.02)
Pain intensity scores (POD3)	11	−0.22 (−0.49 to 0.05)	−0.22 (−0.49 to 0.05)
Total opioid consumption	14	−0.27 (−0.61 to 0.08)	−0.27 (−0.61 to 0.08)
Opioid consumption (POD1)	11	0.11 (−0.07 to 0.29)	0.10 (−0.03 to 0.22)
Opioid consumption (POD2)	10	−0.25 (−0.84 to 0.33)	−0.26 (−0.90 to 0.38)
Opioid consumption (POD3)	10	−0.40 (−0.92 to 0.12)	−0.51 (−1.05 to 0.02)
Total adverse events	26	1.05 (0.91 to 1.20)	1.04 (0.92 to 1.18)

No., number; POD1, postoperative day 1; POD2, postoperative day 2; POD3, postoperative day 3; SWD, standardized mean differences; RR, risk ratio; CI, confidence interval.

Meta-regression analyses were conducted for primary outcome measures that demonstrated significant heterogeneity. The analyses considered several covariates, including surgical procedures, LB dosages, administration techniques, and interventions in the control group. The results of the meta-regression indicated that the type of surgical procedure was a major contributor to the observed significant heterogeneity in pooled pain intensity scores (Table 3). However, no significant sources of heterogeneity were detected for the remaining outcome measures.

**Table 3:** Meta-regression analysis of potential sources of heterogeneity.

Outcomes	Factors	Coefficient	SE	*P*-value	95% CI
Pain intensity scores (POD1)	Surgical procedure	0.54	0.24	0.03	0.07, 1.02
	LB dosage	0.47	0.49	0.33	−0.48, 1.42
	LB administration	−0.57	0.54	0.29	−1.62, 0.49
	Control intervention	0.01	0.27	0.98	−0.52, 0.54
Pain intensity scores (POD2)	Surgical procedure	0.17	0.08	0.03	0.02, 0.32
	LB dosage	0.17	0.23	0.45	−0.28, 0.62
	LB administration	−0.39	0.21	0.07	−0.81, 0.03
	Control intervention	0.07	0.14	0.62	−0.21, 0.35
Pain intensity scores (POD3)	Surgical procedure	9.02	3.34	0.01	2.46, 15.57
	LB dosage	9.61	10.60	0.37	−11.18, 30.39
	LB administration	−11.47	10.43	0.27	−31.91, 8.97
	Control intervention	0.51	6.09	0.93	−11.44, 12.45
Total opioid consumption	Surgical procedure	4.08	5.70	0.47	−7.09, 15.25
	LB dosage	1.45	14.60	0.92	−27.17, 30.06
	LB administration	−3.75	15.43	0.81	−33.99, 26.50
	Control intervention	3.11	8.36	0.71	−13.27, 19.49
Opioid consumption (POD2)	Surgical procedure	7.49	6.63	0.26	−5.51, 20.50
	LB dosage	−14.52	13.45	0.28	−40.87, 11.84
	LB administration	7.42	14.42	0.61	−20.85, 35.68
	Control intervention	6.64	9.32	0.48	−11.63, 24.91
Opioid consumption (POD3)	Surgical procedure	6.56	7.19	0.36	−7.54, 20.65
	LB dosage	−7.92	14.93	0.60	−37.19, 21.34
	LB administration	7.92	14.93	0.60	−21.34, 37.19
	Control intervention	3.58	10.71	0.74	−17.41, 24.57

SE, standard error; CI, confidence intervals; LB, liposomal bupivacaine; POD1, postoperative day 1; POD2, postoperative day 2; POD3, postoperative day 3.

Publication bias is a major factor that may compromise the validity of findings in systematic reviews and meta-analyses. However, when fewer than 10 studies are included in a meta-analysis, it is challenging to reliably assess the sources of funnel plot asymmetry (65). Therefore, in the present study, Egger's test was applied to evaluate potential publication bias. The results demonstrated significant publication bias for several outcome measures, including pain intensity scores on POD3, opioid consumption on POD1, total adverse events, nausea/vomiting, and pruritus (Table 4).

**Table 4:** Egger's test for the included outcome indicators.

Outcomes	No. of included study	Beta 1	SE	*z*-value	*P*-value	Conclusion
Pain intensity scores (POD1)	18	−0.32	0.87	−0.37	0.71	No significant publication bias
Pain intensity scores (POD2)	14	3.38	2.98	1.14	0.26	No significant publication bias
Pain intensity scores (POD3)	11	−7.05	0.99	−7.14	0.00	Significant publication bias
Total opioid consumption	14	−0.30	0.91	−0.33	0.74	No significant publication bias
Opioid consumption (POD1)	11	1.36	0.62	2.20	0.03	Significant publication bias
Opioid consumption (POD2)	10	−2.75	4.35	−0.63	0.53	No significant publication bias
Opioid consumption (POD3)	10	−5.14	3.20	−1.60	0.11	No significant publication bias
Patient satisfaction	7	−1.11	1.73	−0.64	0.52	No significant publication bias
Length of hospital stay	11	1.22	1.01	1.21	0.23	No significant publication bias
Total adverse events	26	−3.42	0.39	−8.80	0.00	Significant publication bias
Nausea/vomiting	11	−3.35	1.16	−2.90	0.00	Significant publication bias
Constipation	5	−2.91	6.25	−0.47	0.64	No significant publication bias
Pruritus	8	−2.06	1.00	−2.07	0.04	Significant publication bias
Dizziness	6	−2.78	1.87	−1.49	0.14	No significant publication bias

No., number; POD1, postoperative day 1; POD2, postoperative day 2; POD3, postoperative day 3; SE, standardized error.

## 4 Discussion

Perioperative pain management in orthopedic surgery has always been a significant clinical concern for orthopedic medical teams. Over the years, multimodal analgesia has played an active role in perioperative pain control following open orthopedic surgeries. Guided by the Cochrane Handbook, the present work aims to generate robust evidence for perioperative pain management in open orthopedic surgeries. This will be achieved through comprehensive electronic database searches and the inclusion of a higher number of high-quality RCTs that provide insights into the safety and efficacy of local anesthetics (LB) in these surgical procedures. The findings of this meta-analysis are as follows: (1) Since 2015, the use of LB in orthopedic surgery has been primarily in the United States, with peak popularity between 2016 and 2018, globally. (2) The analgesic efficacy of LB may differ among various types of open orthopedic surgeries. Subgroup analyses in the present study demonstrated that LB significantly reduced pain intensity scores on POD1 and POD2 in patients undergoing TKA, as well as on POD2 and POD3 in patients with bunion deformities undergoing DMO. (3) LB does not increase the incidence of postoperative complications, but it does not effectively reduce opioid consumption.

LB employs liposomal technology to deliver drugs directly and precisely to the surgical site, enabling a gradual release process that significantly enhances the bioavailability of the medication and offers consistent analgesic effects lasting up to 72 h (66, 67). Therefore, the primary advantage of LB lies in its long-acting postoperative analgesia. Our study demonstrated that pain intensity scores on POD2 were significantly lower in the LB group than in the control group, whereas pain intensity scores on POD1 showed a trend toward statistical significance. These findings indicated that LB may offer superior analgesic effects compared with standard treatment during this early postoperative period. However, studies (55, 58, 60) observing short-term (up to 48 or 72 h postoperative) pain scores did not reveal significant differences in pain scores compared to other drugs.

Currently, the effectiveness of LB in providing prolonged pain relief in open orthopedic surgeries remains uncertain. A recent single-center, double-blind, RCT (68) assessed the analgesic effects of LB in patients undergoing unilateral primary TKA. The study demonstrated that incorporating LB into a multimodal analgesic regimen, including a adductor canal block and periarticular infiltration, resulted in sustained pain relief and superior control of activity-related pain. In contrast, our study found that LB reduced pain intensity scores on POD1 and POD2 in TKA patients. However, previous research (19) reported contradictory findings, showing that LB did not significantly reduce postoperative pain compared to conventional analgesic approaches in TKA patients.

Furthermore, a recent study (69) evaluating the analgesic efficacy of LB vs. conventional anesthetic therapy in patients undergoing hand or wrist surgery reported that, compared with standard bupivacaine, LB did not significantly reduce postoperative pain or opioid consumption, nor did it improve patient-reported analgesic satisfaction. In lumbar fusion surgery, the combination of LB with retrolaminar block has been shown to effectively reduce pain within the first 72 h postoperatively (70). However, the use of ultrasound-guided erector spinae plane block combined with LB demonstrated only a modest advantage in alleviating postoperative pain and improving quality of recovery after lumbar fusion, and these benefits may be of limited clinical significance (71).

In the present study, subgroup analysis revealed that the type of surgery was a major contributor to the substantial heterogeneity observed in postoperative pain intensity scores. Nevertheless, the clinical outcomes of LB administration within the same surgical procedure have been inconsistent (19, 68, 70, 71). This discrepancy is largely attributable to differences in LB administration techniques and the analgesic regimens employed in control groups across studies. Furthermore, interpreting findings from studies that combine local anesthetics with adrenergic agents, nonsteroidal anti-inflammatory drugs, or morphine remains challenging (19). Pharmacokinetic studies (72) revealed that the first peak of LB occurred within 1 h after injection, with a second peak occurring 12–36 h later. This provides a reasonable explanation for the findings of our study. However, due to the short follow-up period in the included studies, the analgesic effect of LB after 72 h could not be observed and requires further validation.

Theoretically, opioid consumption is negatively correlated with pain intensity (67). A meta-analysis (19) on the perioperative use of LB in TKA reported that the morphine consumption in the LB group was similar to that of the control group on POD 0, POD 1, and POD 3; however, the rescue dose of morphine in the LB group was lower before POD 2. A recent RCT [4] also found that retrolaminar block combined with LB did not reduce opioid consumption during the perioperative period of lumbar fusion surgery. However, ultrasound-guided erector spinae block combined with LB demonstrated a more significant reduction in opioid consumption during lumbar fusion surgery compared to local infiltration of LB (71). Our study did not find any differences between the two groups in terms of opioid consumption. The variations in surgical techniques, anesthesia methods, and interventions in the control group may have contributed favorably to these findings. A recent network meta-analysis (73) exploring the optimal analgesic strategy for TSA reported that the LB group had the lowest total postoperative morphine consumption. This is primarily due to the gradual and sustained release process of LB inflation, which mainly prolongs the duration of anesthesia (59).

Meanwhile, a study (19) reported adverse events such as nausea or vomiting associated with LB, but our study did not make the same finding. This may be related to the fact that the application dose of LB included in the current study was always the minimum effective dose ( ≤ 266 mg). Previous study (72) has shown that the incidence of adverse events with a dose of LB ≤ 266 mg was significantly lower than >266 mg.

To our best knowledge, the current work involved the most significant number of RCTs and comprehensively analyzed outcome measures among meta-analyses on similar topics. However, this study has certain limitations. (1) Although our study only included RCTs, several included studies may still have random errors, selection biases, and other issues that could affect the reliability and validity of the findings. Moreover, a relatively high number of studies were rated as having a high or unclear risk of bias, which could directly lead to overestimation and exaggeration, making the conclusions unreliable. Some high-quality studies could not be fully included in the analysis due to a lack of relevant data or incomplete data, potentially introducing biases into the results. (2) Despite our efforts to search relevant literature across multiple databases, some valuable data from unpublished or gray literature may not have been incorporated into this study. Moreover, certain data may not be fully utilized due to limitations or missing information in the original studies, which could also affect the study's results. (3) The included studies in the present work have exhibited significant heterogeneity, mainly due to differences in factors such as the administration methods, drug concentrations, and types of surgeries used in the various studies. These factors could influence the analgesic efficacy and safety of LB. While subgroup analyses and meta-regression were performed to explore sources of heterogeneity, some subgroups had few studies with small sample sizes, which may undermine the clinical relevance and stability of the results. Hence, clinical translation of these findings should be cautious. (4) LB has been shown to provide significant analgesia for up to 72 h post-surgery, which is a key advantage in postoperative pain management. Most studies included in the present work assessed postoperative pain primarily within the first 72 h, reflecting the pharmacologic profile of LB—which is designed to provide extended analgesia mainly within this time frame. However, longer-term data were not available, then we were unable to evaluate analgesic outcomes in the longer follow-up.

## 5 Conclusion

In conclusion, our study indicates that the use of LB in open orthopedic surgeries does not increase the incidence of related complications. However, compared to other conventional medications (bupivacaine, ropivacaine, etc.), LB does not show a significant advantage in perioperative pain management, nor does it significantly reduce opioid consumption. Due to the heterogeneity among the studies, more high-quality RCTs are needed to confirm this conclusion further. Furthermore, we should pay more attention to the long-term safety of LB to ensure its reliability in practical applications.

## Data Availability

The original contributions presented in the study are included in the article/Supplementary material, further inquiries can be directed to the corresponding authors.

## References

[1] Harder T Harder J Baum G Cox C Harder J Hernandez E . Antineuropathic pain management after orthopedic surgery: a systematic review. Orthop Rev. (2024) 16:93012. doi: 10.52965/001c.93012 PMC10950197 38505138

[2] Gerbershagen HJ Aduckathil S van Wijck AJM Peelen LM Kalkman CJ Meissner W. Pain intensity on the first day after surgery: a prospective cohort study comparing 179 surgical procedures. Pain Med. (2013) 118:934–44. doi: 10.1097/ALN.0b013e31828866b3 23392233

[3] El-Boghdadly K Levy NA Fawcett WJ Knaggs RD Laycock H Baird E . Peri-operative pain management in adults: a multidisciplinary consensus statement from the Association of Anaesthetists and the British Pain Society. Anaesthesia. (2024) 79:1220–36. doi: 10.1111/anae.16391 39319373

[4] Walker EMK Bell M Cook TM Grocott MPW Moonesinghe SR. Patient reported outcome of adult perioperative anaesthesia in the United Kingdom: a cross-sectional observational study. Br J Anaesth. (2016) 117:758–66. doi: 10.1093/bja/aew381 27956674

[5] Wylde V Rooker J Halliday L Blom A. Acute postoperative pain at rest after hip and knee arthroplasty: severity, sensory qualities and impact on sleep. Orthop Traumatol Surg Res. (2011) 97:139–44. doi: 10.1016/j.otsr.2010.12.003 21388906

[6] Glare P Aubrey KR Myles PS. Transition from acute to chronic pain after surgery. Lancet. (2019) 393:1537–46. doi: 10.1016/S0140-6736(19)30352-6 30983589

[7] Drevet S Boussat B Grevy A Brevet A Olive F Richard M . Perioperative pain management intervention in older patients with hip fracture in an orthogeriatric unit. A controlled before/after study assessing an audit and feedback intervention (PAIN-AGE). BMC Geriatr. (2024) 24:735. doi: 10.1186/s12877-024-05282-w 39237920 PMC11375881

[8] Mahmoud A Abuelazm M Ashraf Salah Ahmed A Elshinawy M Ashour T Abugdida M . Early perioperative versus postoperative meloxicam for pain control in patients undergoing orthopedic surgery: a systematic review and Meta-analysis of randomized controlled trials. Curr Med Res Opin. (2023) 39:113–22. doi: 10.1080/03007995.2022.2135837 36245362

[9] Trenchfield D Fras S McCurdy M Narayanan R Lee Y Issa T . Opioid prescription trends among orthopaedic, primary care, and pain management providers in spine surgery patients. J Am Acad Orthop Surg. (2024) 32:e1252–9. doi: 10.5435/JAAOS-D-24-00167 39186611

[10] Ivanova JI Birnbaum HG Schiller M Kantor E Johnstone BM Swindle RW. Real-world practice patterns, health-care utilization, and costs in patients with low back pain: the long road to guideline-concordant care. Spine J. (2011) 11:622–32. doi: 10.1016/j.spinee.2011.03.017 21601533

[11] Nguyen H V Mital S Bugden S McGinty EE. British Columbia's safer opioid supply policy and opioid outcomes. JAMA Intern Med. (2024) 184:256–64. doi: 10.1001/jamainternmed.2023.7570 PMC10792500 38227344

[12] Vowles KE McEntee ML Julnes PS Frohe T Ney JP Van Der Goes DN. Rates of opioid misuse, abuse, and addiction in chronic pain: a systematic review and data synthesis. Pain. (2015) 156:569–76. doi: 10.1097/01.j.pain.0000460357.01998.f1 25785523

[13] KC B Alrasheedy AA Mohamed Ibrahim MI Paudyal V Christopher CM Shrestha S . Combatting opioid misuse, overuse and abuse: a systematic review of pharmacists' services and outcomes. Pain Manag. (2024) 14:519–29. doi: 10.1080/17581869.2024.2411930 PMC11728331 39439259

[14] Nguyen A Grape S Gobbetti M Albrecht E. The postoperative analgesic efficacy of liposomal bupivacaine versus long-acting local anaesthetics for peripheral nerve and field blocks: a systematic review and meta-analysis, with trial sequential analysis. Eur J Anaesthesiol. (2023) 40:624–35. doi: 10.1097/EJA.0000000000001833 PMC10860892 37038770

[15] Lee JK Greenberg S Wixson R Heshmat C Locke A Daniel T . Liposomal bupivacaine interscalene blocks demonstrate a greater proportion of total shoulder arthroplasty patients with clinically tolerable pain: a retrospective quality improvement study of 491 patients. J ISAKOS. (2024) 9:9–15. doi: 10.1016/j.jisako.2023.10.007 37866512

[16] Alter TH Liss FE Ilyas AM. A prospective randomized study comparing bupivacaine hydrochloride versus bupivacaine liposome for pain management after distal radius fracture repair surgery. J Hand Surg. (2017) 42:1003–8. doi: 10.1016/j.jhsa.2017.08.022 28969978

[17] Kang KK Voyvodic L Komlos D Swiggett S Ng MK. Liposomal bupivacaine does not decrease postoperative pain in patients with Iintracapsular femoral neck fracture treated with Hemiarthroplasty: HEAT - a randomized, controlled trial. J Bone Joint Surg. (2024) 106:1957–62. doi: 10.2106/JBJS.23.01344 39292808

[18] Chung AS Crandall D Revella J Adeniyi B Chang YHH Chang MS. Does local administration of liposomal bupivacaine reduce pain and narcotic consumption in adult spinal deformity surgery? Global Spine J. (2021) 11:896–902. doi: 10.1177/2192568220931053 PMC8258814 32677519

[19] Chen JJ Wu YC Wang JS Lee CH. Liposomal bupivacaine administration is not superior to traditional periarticular injection for postoperative pain management following total knee arthroplasty: a meta-analysis of randomized controlled trials. J Orthop Surg Res. (2023) 18:206. doi: 10.1186/s13018-023-03699-4 PMC10018851 36922892

[20] Daher M Singh M Nassar JE Casey JC Callanan TC Diebo BG . Liposomal bupivacaine reduces postoperative pain and opioids consumption in spine surgery: a meta-analysis of 1,269 patients. Spine J. (2024) 25:411–8. doi: 10.1016/j.spinee.2024.10.013 39491751

[21] Hyland SJ Deliberato DG Fada RA Romanelli MJ Collins CL Wasielewski RC. Liposomal bupivacaine versus standard periarticular injection in total knee arthroplasty with regional anesthesia: a prospective randomized controlled trial. J Arthroplasty. (2019) 34:488–94. doi: 10.1016/j.arth.2018.11.026 30554925

[22] Stundner O Hoerner E Zhong H Poeran J Liu J Illescas A . Trends of liposomal bupivacaine utilization in major lower extremity total joint arthroplasty in the USA: a population-based study. Reg Anesth Pain Med. (2024) 49:139–43. doi: 10.1136/rapm-2023-104784 37567594

[23] Higgins J Thomas J Chandler J Cumpston M Li T Page M . Cochrane Handbook for Systematic Reviews of Interventions Version 6.5. Cochrane (2024). Available online at: www.training.cochrane.org/handbook (Accessed August 2024)

[24] Page MJ McKenzie JE Bossuyt PM Boutron I Hoffmann TC Mulrow CD . The PRISMA 2020 statement: an updated guideline for reporting systematic reviews. BMJ. (2021) 372:n71. doi: 10.1136/bmj.n71 PMC8005924 33782057

[25] Sterne JAC White IR Carlin JB Spratt M Royston P Kenward MG . Multiple imputation for missing data in epidemiological and clinical research: potential and pitfalls. BMJ. (2009) 338:b2393. doi: 10.1136/bmj.b2393 PMC2714692 19564179

[26] Hughes D Judge C Murphy R Loughlin E Costello M Whiteley W . Association of blood pressure lowering with Incident dementia or cognitive impairment: a systematic review and meta-analysis. JAMA. (2020) 323:1934–44. doi: 10.1001/jama.2020.4249 PMC7237983 32427305

[27] Higgins JPT Thompson SG. Quantifying heterogeneity in a meta-analysis. Stat Med. (2002) 21:1539–58. doi: 10.1002/sim.1186 12111919

[28] Choi SW Lam DMH. Heterogeneity in meta-analyses. Comparing apples and oranges? Anaesthesia. (2017) 72:532–4. doi: 10.1111/anae.13832 28213890

[29] Bramlett K Onel E Viscusi ER Jones K. A randomized, double-blind, dose-ranging study comparing wound infiltration of DepoFoam bupivacaine, an extended-release liposomal bupivacaine, to bupivacaine HCl for postsurgical analgesia in total knee arthroplasty. Knee. (2012) 19:530–6. doi: 10.1016/j.knee.2011.12.004 22285545

[30] Schroer WC Diesfeld PG LeMarr AR Morton DJ Reedy ME. Does extended-release liposomal bupivacaine better control pain than bupivacaine after total knee arthroplasty (TKA)? A prospective, randomized clinical trial. J Arthroplasty. (2015) 30:64–7. doi: 10.1016/j.arth.2015.01.059 26117072

[31] Jain RK Porat MD Klingenstein GG Reid JJ Post RE Schoifet SD. Liposomal bupivacaine and periarticular injection are not superior to single-shot intra-articular injection for pain control in total knee arthroplasty. J Arthroplasty. (2016) 31:22–5. doi: 10.1016/j.arth.2016.03.036 27113945

[32] Snyder MA Scheuerman CM Gregg JL Ruhnke CJ Eten K. Improving total knee arthroplasty perioperative pain management using a periarticular injection with bupivacaine liposomal suspension. Arthroplast Today. (2016) 2:37–42. doi: 10.1016/j.artd.2015.05.005 PMC4957154 28326395

[33] Schwarzkopf R Drexler M Ma MW Schultz VM Le KT Rutenberg TF . Is there a benefit for liposomal bupivacaine compared to a traditional periarticular injection in total knee arthroplasty patients with a history of chronic opioid use? J Arthroplasty. (2016) 31:1702–5. doi: 10.1016/j.arth.2016.01.037 26897490

[34] Collis PN Hunter AM Vaughn MDD Carreon LY Huang J Malkani AL. Periarticular injection after total knee arthroplasty using liposomal bupivacaine vs a modified ranawat suspension: a prospective, randomized study. J Arthroplasty. (2016) 31:633–6. doi: 10.1016/j.arth.2015.09.025 26482684

[35] Okoroha KR Lynch JR Keller RA Korona J Amato C Rill B . Liposomal bupivacaine versus interscalene nerve block for pain control after shoulder arthroplasty: a prospective randomized trial. J Shoulder Elbow Surg. (2016) 25:1742–8. doi: 10.1016/j.jse.2016.05.007 27422692

[36] Smith EB Kazarian GS Maltenfort MG Lonner JH Sharkey PF Good RP. Periarticular liposomal bupivacaine injection versus intra-articular bupivacaine infusion catheter for analgesia after total knee arthroplasty. J Bone Joint Surg Am Vol. (2017) 99:1337–44. doi: 10.2106/JBJS.16.00571 28816893

[37] Alijanipour P Tan TL Matthews CN Viola JR Purtill JJ Rothman RH . Periarticular injection of liposomal bupivacaine offers no benefit over standard bupivacaine in total knee arthroplasty: a prospective, randomized, controlled trial. J Arthroplasty. (2017) 32:628–34. doi: 10.1016/j.arth.2016.07.023 27667533

[38] DeClaire JH Aiello PM Warritay OK Freeman DC. Effectiveness of bupivacaine liposome injectable suspension for postoperative pain control in total knee arthroplasty: a prospective, randomized, double blind, controlled study. J Arthroplasty. (2017) 32:S268–71. doi: 10.1016/j.arth.2017.03.062 28478185

[39] Barrington JW Emerson RH Lovald ST Lombardi AV Berend KR. No difference in early analgesia between liposomal bupivacaine injection and intrathecal morphine after TKA. Clin Orthop Relat Res. (2017) 475:94–105. doi: 10.1007/s11999-016-4931-z PMC5174037 27339124

[40] Amundson AW Johnson RL Abdel MP Mantilla CB Panchamia JK Taunton MJ . A three-arm randomized clinical trial comparing continuous femoral plus single-injection sciatic peripheral nerve blocks versus periarticular injection with ropivacaine or liposomal bupivacaine for patients undergoing total knee arthroplasty. Anesthesiology. (2017) 126:1139–50. doi: 10.1097/ALN.0000000000001586 28234636

[41] Davidovitch R Goch A Driesman A Konda S Pean C Egol K. The use of liposomal bupivacaine administered with standard bupivacaine in ankle fractures requiring open reduction internal fixation: a single-blinded randomized controlled trial. J Orthop Trauma. (2017) 31:434–9. doi: 10.1097/BOT.0000000000000862 28430722

[42] Vandepitte C Kuroda M Witvrouw R Anne L Bellemans J Corten K . Addition of liposome bupivacaine to bupivacaine HCl versus bupivacaine HCl alone for interscalene brachial plexus block in patients having major shoulder surgery. Reg Anesth Pain Med. (2017) 42:334–41. doi: 10.1097/AAP.0000000000000560 28157791

[43] Sabesan VJ Shahriar R Petersen-Fitts GR Whaley JD Bou-Akl T Sweet M . A prospective randomized controlled trial to identify the optimal postoperative pain management in shoulder arthroplasty: liposomal bupivacaine versus continuous interscalene catheter. J Shoulder Elbow Surg. (2017) 26:1810–7. doi: 10.1016/j.jse.2017.06.044 28844420

[44] Namdari S Nicholson T Abboud J Lazarus M Steinberg D Williams G. Randomized controlled trial of interscalene block compared with injectable liposomal bupivacaine in shoulder arthroplasty. J Bone Joint Surg Am Vol. (2017) 99:550–6. doi: 10.2106/JBJS.16.00296 28375887

[45] Abildgaard JT Lonergan KT Tolan SJ Kissenberth MJ Hawkins RJ Washburn R . Liposomal bupivacaine versus indwelling interscalene nerve block for postoperative pain control in shoulder arthroplasty: a prospective randomized controlled trial. J Shoulder Elbow Surg. (2017) 26:1175–81. doi: 10.1016/j.jse.2017.03.012 28479257

[46] Johnson RL Amundson AW Abdel MP Sviggum HP Mabry TM Mantilla CB . Continuous posterior lumbar plexus nerve block versus periarticular injection with ropivacaine or liposomal bupivacaine for total hip arthroplasty: a three-arm randomized clinical trial. J Bone Joint Surg Am Vol. (2017) 99:1836–45. doi: 10.2106/JBJS.16.01305 29088038

[47] Zlotnicki JP Hamlin BR Plakseychuk AY Levison TJ Rothenberger SD Urish KL. Liposomal bupivacaine vs plain bupivacaine in periarticular injection for control of pain and early motion in total knee arthroplasty: a randomized, prospective study. J Arthroplasty. (2018) 33:2460–4. doi: 10.1016/j.arth.2018.03.014 PMC6054911 29656977

[48] Mont MA Beaver WB Dysart SH Barrington JW Del Gaizo DJ. Local infiltration analgesia with liposomal bupivacaine improves pain scores and reduces opioid use after total knee arthroplasty: results of a randomized controlled trial. J Arthroplasty. (2018) 33:90–6. doi: 10.1016/j.arth.2017.07.024 28802777

[49] Suarez JC Al-Mansoori AA Kanwar S Semien GA Villa JM McNamara CA . Effectiveness of novel adjuncts in pain management following total knee arthroplasty: a randomized clinical trial. J Arthroplasty. (2018) 33:S136–41. doi: 10.1016/j.arth.2018.02.088 29628196

[50] Namdari S Nicholson T Abboud J Lazarus M Steinberg D Williams G. Interscalene block with and without intraoperative local infiltration with liposomal bupivacaine in shoulder arthroplasty: a randomized controlled trial. J Bone Joint Surg Am Vol. (2018) 100:1373–8. doi: 10.2106/JBJS.17.01416 30106818

[51] Perets I Walsh JP Mu BH Yuen LC Ashberg L Battaglia MR . Intraoperative infiltration of liposomal bupivacaine vs bupivacaine hydrochloride for pain management in primary total hip arthroplasty: a prospective randomized trial. J Arthroplasty. (2018) 33:441–6. doi: 10.1016/j.arth.2017.09.013 29033152

[52] Brown L Weir T Shasti M Yousaf O Yousaf I Tannous O . The efficacy of liposomal bupivacaine in lumbar spine surgery. Int J Spine Surg. (2018) 12:434–40. doi: 10.14444/5052 PMC6159630 30276103

[53] Schumer G Mann JW Stover MD Sloboda JF Cdebaca CS Woods GM. Liposomal bupivacaine utilization in total knee replacement does not decrease length of hospital stay. J Knee Surg. (2019) 32:934–9. doi: 10.1055/s-0038-1673617 30372781

[54] Brown L Weir T Koenig S Shasti M Yousaf I Yousaf O . Can liposomal bupivacaine be safely utilized in elective spine surgery? Global Spine J. (2019) 9:133–7. doi: 10.1177/2192568218755684 PMC6448202 30984490

[55] Hattrup SJ Chung AS Rosenfeld DM Misra L Koyyalamudi V Ritz ML . Liposomal bupivacaine interscalene nerve block in shoulder arthroplasty is not superior to plain bupivacaine: a double-blinded prospective randomized control trial. J Shoulder Elbow Surg. (2021) 30:587–98. doi: 10.1016/j.jse.2020.09.017 33045330

[56] Ali I Gupta HO Khazzam M Thomas GL Vattigunta S Shi BY . Do local liposomal bupivacaine and interscalene nerve block provide similar pain control after shoulder arthroplasty? A dual-center randomized controlled trial. J Shoulder Elbow Surg. (2021) 30:S145–52. doi: 10.1016/j.jse.2021.04.010 33894365

[57] Ewing M Huff H Heil S Borsheski RR Smith MJ Kim HM. Local Infiltration analgesia versus interscalene block for pain management following shoulder arthroplasty: a prospective randomized clinical trial. J Bone Joint Surg. (2022) 104:1730–7. doi: 10.2106/JBJS.22.00034 35778995

[58] Schumaier A Kloby M Hasselfeld K Grawe B. Interscalene block vs. periarticular liposomal bupivacaine for pain control following reverse shoulder arthroplasty: a randomized trial. J Shoulder Elbow Surg. (2023) 32:1412–9. doi: 10.1016/j.jse.2023.02.137 37003430

[59] Krupp R Smith A Nyland J Mojesky C Perkins D Carreon LY. Liposomal bupivacaine nerve block provides better pain control post-total shoulder arthroplasty than continuous indwelling catheter. Arch Orthop Trauma Surg. (2023) 143:1895–902. doi: 10.1007/s00402-022-04386-5 35235030

[60] Quaye A McAllister B Garcia JR Nohr O Laduzenski SJ Mack L . A prospective, randomized trial of liposomal bupivacaine compared to conventional bupivacaine on pain control and postoperative opioid use in adults receiving adductor canal blocks for total knee arthroplasty. Arthroplasty. (2024) 6:6. doi: 10.1186/s42836-023-00226-y 38297390 PMC10832097

[61] Finkel KJ Walker A Maffeo-Mitchell CL Nissen C Kainkaryam P Sposito J . Liposomal bupivacaine provides superior pain control compared to bupivacaine with adjuvants in interscalene block for total shoulder replacement: a prospective double-blinded, randomized controlled trial. J Shoulder Elbow Surg. (2024) 33:1512–20. doi: 10.1016/j.jse.2023.12.014 38311100

[62] Poonam PBH Bohaczuk S Jinadu S Hong J Echevarria G Lai YH . Comparison of analgesic efficacy of continuous perineural catheter, liposomal bupivacaine, and dexamethasone as an adjuvant for interscalene block in total Shoulder arthroplasty: a triple blinded randomized controlled trial. J Shoulder Elbow Surg. (2024) 34:e81–e92. doi: 10.1016/j.jse.2024.06.014 39197650

[63] Schwartz G Gadsden JC Gonzales J Hutchins J Song J Brady OD . A phase 3 active-controlled trial of liposomal bupivacaine via sciatic nerve block in the popliteal fossa after bunionectomy. J Clin Anesth. (2024) 94:111402. doi: 10.1016/j.jclinane.2024.111402 38340677

[64] Chan TCW Wong JSH Wang F Fang CX Yung CSY Chan MTH . Addition of liposomal bupivacaine to standard bupivacaine versus standard bupivacaine alone in the supraclavicular brachial plexus block: a randomized controlled trial. Anesthesiology. (2024) 141:732–44. doi: 10.1097/ALN.0000000000005035 PMC11389883 38696340

[65] Sterne JA Sutton AJ Ioannidis JP Terrin N Jones DR Lau J . Recommendations for examining and interpreting funnel plot asymmetry in meta-analyses of randomised controlled trials. BMJ. (2011) 343:d4002. doi: 10.1136/bmj.d4002 21784880

[66] Richard BM Newton P Ott LR Haan D Brubaker AN Cole PI . The safety of EXPAREL^®^ (bupivacaine liposome injectable suspension) administered by peripheral nerve block in rabbits and dogs. J Drug Deliv. (2012) 2012:962101. doi: 10.1155/2012/962101 PMC3270427 22363842

[67] Muchintala R Kern N Davis G Ioffreda P Rengifo S Khak M. Periarticular injections with liposomal bupivacaine in comparison with traditional (bupivacaine/ropivacaine) periarticular injections in total hip arthroplasty: a systematic review. SurgiColl. (2024) 2. doi: 10.58616/001c.90487

[68] Yin Z Chen J Zhao T Chen Y Wang B Ma J. Analgesic efficacy of liposomal bupivacaine adductor canal block combined with periarticular infiltration after total knee arthroplasty: a prospective, double-blind, randomized controlled trial. BMC Anesthesiol. (2026) 26:251. doi: 10.1186/s12871-026-03742-x PMC13097951 41820856

[69] Garavito JA Nassar JE Johnson J Singh M Got C Gil JA. The use of liposomal bupivacaine for pain control after hand and wrist surgery: a systematic review and meta-analysis. J Orthop. (2025) 73:213–20. doi: 10.1016/j.jor.2025.12.030 PMC12771307 41503229

[70] Zhang SJ Liu Y Wei YQ Ma XY Wang YB. Efficacy and safety of liposomal bupivacaine versus bupivacaine for retrolaminar block for postoperative analgesia in patients undergoing posterior lumbar interbody fusion: a randomized controlled trial. BMC Anesthesiol. (2026) 26:235. doi: 10.1186/s12871-026-03721-2 PMC13081550 41794684

[71] Niu JY Yang YD Ouyang RN Liu D Zhang Q Qi JL . Comparison of liposomal bupivacaine erector spinae plane block versus wound infiltration for postoperative analgesia and quality of recovery in lumbar fusion surgery: a randomized controlled trial. Eur J Med Res. (2025) 30:883. doi: 10.1186/s40001-025-03174-2 PMC12465786 41013624

[72] Hu D Onel E Singla N Kramer WG Hadzic A. Pharmacokinetic profile of liposome bupivacaine injection following a single administration at the surgical site. Clin Drug Investig. (2013) 33:109–15. doi: 10.1007/s40261-012-0043-z 23229686

[73] Li S Chen W Feng L Guo X. Optimal analgesic regimen for total shoulder arthroplasty: a randomized controlled trial and network meta-analysis. J Orthop Surg Res. (2024) 19:57. doi: 10.1186/s13018-023-04451-8 38216998 PMC10785537

