# Supplementary material for: Nerve block or wound infiltration with liposomal bupivacaine for postoperative pain management in open orthopedic surgery: a meta-analysis of 38 randomized controlled trials involving 3,389 patients
Source: Front Med (Lausanne). 2026 Sep 18;13:1820439. doi: 10.3389/fmed.2026.1820439 (PMC13630734; doi:10.3389/fmed.2026.1820439)
Supplement: Supplementary file 1 [file Table_1.docx]

**Search Strategy**

**Medline via PubMed**

#1: liposomal bupivacaine [All field]

#2: liposome [All field]

#3: exparel [All field]

#4: #1 OR #2 OR #3

#5: orthopedic surgery [All field]

#6: spine [All field]

#7: arthroplasty [All field])

#8: joint replacement [All field]

#9: shoulder surgery [All field]

#10: fracture [All field])

#11: #5 OR #6 OR #7 OR #8 OR #9 OR #10

#12: 01/01/2010 to 30/09/2025 [Date - Publication]

#13: #4 AND #11 AND #12

**Web of Science**

#1 TS=(liposomal bupivacaine OR liposome OR exparel)

#2 TS=(orthopedic surgery OR spine OR arthroplasty OR joint replacement OR shoulder surgery OR fracture)

#3 PY=(2010-2025)

#4 #1 AND #2 AND #3

**Embase**

#1 ‘liposomal bupivacaine’/exp

#2: ‘liposomal bupivacaine’:ab,ti,kw OR ‘liposome’:ab,ti,kw OR ‘exparel’:ab,ti,kw

#3: #1 OR #2

#4: ‘orthopedic surgery’:ab,ti,kw OR ‘spine’:ab,ti,kw OR ‘arthroplasty’:ab,ti,kw OR ‘joint replacement’:ab,ti,kw OR ‘shoulder surgery’:ab,ti,kw OR ‘fracture’:ab,ti,kw

#5: [2010-2025]/py

#6: #3 AND #4 AND #5

**Cochrane Central Register of Controlled Clinical Trials**

#1 “liposomal bupivacaine” OR liposome OR exparel

#2 “orthopedic surgery” OR spine OR arthroplasty OR “joint replacement” OR “shoulder surgery” OR fracture

#3 (#1 AND #2)

**Google Scholar**

(“liposomal bupivacaine” OR liposome OR exparel) AND (“orthopedic surgery” OR spine OR arthroplasty OR “joint replacement” OR “shoulder surgery” OR fracture)
